# Phytoconstituents and Bioactivity of Plants of the Genus *Spiraea* L. (Rosaceae): A Review

**DOI:** 10.3390/ijms222011163

**Published:** 2021-10-16

**Authors:** Vera A. Kostikova, Natalia V. Petrova

**Affiliations:** 1 Central Siberian Botanical Garden, Siberian Branch of Russian Academy of Sciences, 630090 Novosibirsk, Russia; 2 Laboratory Herbarium (TK), Tomsk State University, 634050 Tomsk, Russia; 3 Komarov Botanical Institute, Russian Academy of Sciences, 197376 St. Petersburg, Russia; npetrova@binran.ru

**Keywords:** *Spiraea*, flavonoid, terpenoid, acid, biological activity, biotechnology

## Abstract

The genus *Spiraea* L. belongs to the Rosaceae Juss. family and includes more than 100 species distributed in the temperate zone and subtropical zone of the Northern Hemisphere at the center of species diversity in East Asia. Representatives of the genus are known as ornamental plants with many forms and varieties, are widely used in conventional medicine, and have a high resource potential. This review provides information on the diversity of phenolic compounds (flavonoids, phenolcarboxylic acids, and lignans), terpenoids, alkaloids, steroids, and other classes of secondary metabolites in the species of *Spiraea* worldwide. The article also presents little-known and hard-to-find data published in Russian concerning *Spiraea* phytochemistry. The biological activities of extracts and their fractions and of individual compounds having different types of biological activity (e.g., antioxidant, antibacterial, anti-inflammatory, and antifungal) are discussed. Data about biotechnological research on representatives of the genus *Spiraea* are presented too. The analysis of the literature showed that further chemical and pharmacological studies on *Spiraea* plants are quite promising.

## 1. Introduction

The family Rosaceae Juss. is represented by three subfamilies, 16 tribes, 88–100 genera, and ~3000 species [1,2]. It is one of the most economically important families. Rosaceae includes the most common fruit and berry crops (e.g., apple, strawberry, pear, peach, plum, almond, raspberry, and cherry) and essential-oil crops (e.g., rose and almond). In addition to commercial species serving as important food sources, Rosaceae includes many plants with ornamental and therapeutic properties [3]. Species of the genus *Spiraea* L. are affiliated with these plants.

**Figure 1:**
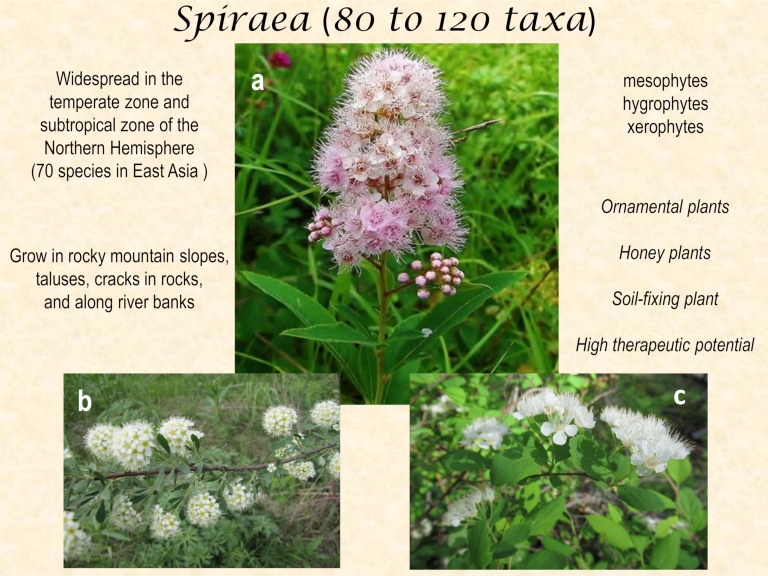
Plants of the genus *Spiraea* (some representatives: (**a**) *S. salicifolia*; (**b**) *S. crenata*; and (**c**) *S. ussuriensis*).

*Spiraea* species (spireas) are deciduous shrubs 0.15 to 2.50 m in height, with alternate, simple, estipulate leaves, perfectly bisexual or dioecious flowers that are white, light or dark pink, red to purple, in simple or compound inflorescences, with a cup-shaped or bell-shaped hypanthium, five sepals, five petals, five carpels, usually stamens, and free follicles that dehisce along the inner suture (for more detailed description see refs. [4,5]) (Figure 1). This taxonomically complex genus belongs to the subfamily Amygdaloideae (formerly Spiraeoideae), tribe Spiraeeae, and contains, according to various estimates, 80 to 120 taxa [1,6]. Phylogenetic relationships within the genus have not been studied sufficiently, and evolutionary patterns for morphological characteristics remain unclear [7]. Due to high polymorphism, spirea has been subdivided by various authors into subgenera, sections, and series [8,9,10].

Spireas are widespread in the temperate zone and subtropical zone of the Northern Hemisphere. According to O.A. Svyazeva [11], the geographic range of the genus *Spiraea* covers most of the former Soviet Union, from the Carpathians to the shores of the Pacific Ocean and from Chukotka to Mongolia and China, and can be characterized as a continuous range with two islands: in the Caucasus and Kopet-Dag. Many spireas are widespread (*S. media*, *S. salicifolia*, and *S. hypericifolia*), while others are narrow endemics (*S. baldschuanica* and *S. pilosa*). The highest species diversity of the genus is observed in East Asia, mainly in China, where 70 species of *Spiraea* are distinguished [5]. Additionally, substantial diversity is found in the Himalayas, Central Asia, the Far East of Russia, and the south of Central Europe. Eight species occur in the United States and Canada [12,13]. Spireas usually grow in open spaces: rocky mountain slopes, taluses, cracks in rocks, and along riverbanks. Representatives of the genus *Spiraea* include mesophytes, hygrophytes, and xerophytes [11]. Spireas are some of the most low-maintenance shrubs (tolerant of diverse conditions), which are valued primarily for their decorative use. Possessing a wide variety of shapes and sizes of the bush, flowering abundance levels, and colors and shapes of inflorescences and leaves, many *Spiraea* species are widely used in landscaping. Nonetheless, the value of these species is not limited to their decorative effect. Many spireas are honey plants, and some spireas can also serve as a soil-fixing plant (Figure 1).

The ethnomedicinal uses of spireas have been documented in North America, Russia, and Asia. Spireas are employed as effective therapeutics for inflammation and malaria [14]. In traditional Chinese medicine, young leaves, fruits, and roots of *S. japonica* and its varieties are used as a diuretic, detoxifier, and pain reliever as well as to treat coughs, headaches, and toothaches [15,16]. Wood oil obtained after burning *S. canescens* is utilized against skin problems [17]. A decoction of *S. betulifolia* has been used as an analgesic and against menstrual pain, heavy or prolonged menstruation, kidney failure, rashes, colds, and abdominal pains by Native Americans [18,19]. *S. salicifolia* is employed in Russian, folk Mongolian, and Tibetan medicine. Decoctions and infusions are prescribed against gastrointestinal diseases, rheumatoid arthritis, helminthiases, gynecological diseases, and diabetes [20,21]. The validity of the medicinal use of spireas in folk medicine has been confirmed by modern scientific experiments [22,23,24].

By the mid-1990s, information on the chemical composition of spireas had been scarce. The available data were mainly about the alkaloid content of *S. japonica* species. After the publication of a review article by X. Hao et al. [25] about alkaloids of the *S. japonica* complex, more than 10 new compounds have been isolated and characterized, and some of the previously discovered substances have been found in other varieties of *S. japonica*. The composition of essential oils has been determined in three *Spiraea* species [26,27,28]. By the early 2000s, spireas had begun to attract the attention of chemists and pharmacologists, and active studies have continued in the recent decades. For example, new flavonoid glycosides have been discovered that have been isolated only from spireas so far [29,30,31,32,33]. New neolignane glycosides, monoterpene acylglycosides, megastigmane glycosides, and other compounds have been isolated from the extracts of various *Spiraea* species [31,34,35,36]. To date, other similarly highly active substances have been found in spireas, and their biological activities have been investigated in a few studies. We are not aware of any overview of the literature on the chemical composition and biological activities of *Spiraea* species. The aim of this paper is to present the first English-language comprehensive review on plants of the genus *Spiraea* by summarizing phytochemical, pharmacological, and biotechnological research in this field.

## 2. Methods

Scientific literature was searched in various databases, including PubMed, Scopus, Google, Google Scholar, e-Library, and Web of Science, by means of “*Spiraea*” as a keyword. All articles published from 1933 to May 2021 in English—as well as in Russian, Chinese, Korean, Turkish, Ukrainian, and German with English abstracts—were found. Those that did not address the phytochemistry, biological activity, and/or ethnopharmacology of *Spiraea* species were discarded. Chemical structures of the phytocomponents were found in the PubChem database, and ChemDraw 19.0 was used to draw selected structures.

## 3. Chemical Components and Biological Activity of the Isolated Substances

Various classes of natural compounds have been found in spireas, and flavonoids and their derivatives are the most studied at present. In addition, lignans, terpenes, and other compounds have been detected in these plants. The structures of all the isolated compounds have been determined by chemical and modern spectroscopic methods. As far as we know, more than 80 alkaloids characteristic of the *S. japonica* complex, six new flavonoid glycosides, one lignan, one neolignan glycoside, four monoterpene acylglycosides, and one megastigmane glycoside have so far been found only in spireas. Most studies on the chemical composition of spireas have been performed on plant samples (leafy shoots, fresh or dry leaves, and extremely rarely, flowers or underground parts) from Siberia and the Far East of Russia as well as Japan and China.

### 3.1. Aromatic Compounds

Spireas, just as other flowering plants, produce phenolic compounds, most of which are flavonoids [37]. The flavonoid content of leaves varies within individual species; for example, in *S. salicifolia*, the concentration ranges from 46.24 to 120 mg/g (dry weight), whereas in *S. dahurica*, it does not exceed 43.63 mg/g [37,38].

Most of the flavonoids of spireas are flavonols, flavones, and catechins: derivatives of quercetin, kaempferol, apigenin, luteolin, and catechin (Table 1). 

As a result of chemotaxonomic research on spireas and subsequent studies, scientists have discovered flavones called apigenin (**1**) and luteolin (**7**) and their C-5 and C-7 *O*-glucosides and C-glucosides: 8-C-glucosides (**4**) and apigenin-6,8-di-C-glucoside (**5**).

Among flavonols, there are only numerous (more than 25) derivatives of quercetin and kaempferol. The diversity of flavonol structures is mainly determined by the presence of a glycoside residue at position C-3 (extremely rarely at C-4′ or C-6″). The most common carbohydrate residues are glucose and galactose, and less frequently, rhamnose; caffeic acid or glucuronic acid or rutin can also serve as substituents.

It should be noted that most flavonols, for example, quercetin (**22**), kaempferol (**10**), isorhamnetin (**28**), hyperoside (**37**), quercetrin (**39**), rutin (**45**), and others, that have been found in spireas are quite common in the plant kingdom, whereas some compounds have so far been found only in spireas. For instance, in the aerial part of *S. prunifolia* var. *simpliciflora*, three new flavonoid glycosides have been identified, named prunifolianosides A–C (**50**, **20**, and **32**, respectively) [31]. In 2017, D.N. Olennikov and N.I. Kashchenko found 37 compounds in flowering shoots of *S. salicifolia*, including a new quercetin glycoside with the structural formula quercetin-3-*O*-[6″-(4′′′-hydroxy-2′′′-methylenebutyroyl)]-β-D-glucopyranoside, named spireasalicin (**49**) by those authors [32]. In 2018, from an ethyl acetate fraction of *S. salicifolia* flowering shoots, D.N. Olennikov and N.K. Chirikova isolated rhamnetin-3-*O*-(6″-*O*-p-coumaroyl)-β-D-glucopyranoside and rhamnetin-3-*O*-(6″-*O*-caffeoyl)-β-D-glucopyranoside, named spiraearhamnins A and B (**26**, **27**), respectively [33]. It should be noted that acylated glycosides of rhamnetin are a very rare family of flavonoids, which until now has included only two representatives found in *Rhamnus petiolaris* Boiss. (Rhamnaceae) and *Campylospermum calanthum* (Gilg.) Farron. (Ochnaceae) [69,70]. Three new dimeric flavonols, namely, sparins A–C (**6**, **54**, **55**), have been isolated from the CHCl_3_ subfraction of an EtOH extract of the *S. brahuica* whole plant [30]. A new flavonoid called 6′-*O*-(4″-methoxy-*trans*-cinnamoyl)-kaempferol-3-β-D-glucopyranoside (**21**) has been isolated from *S*. *canescens* [29].

Flavonoids are a large class of chemical compounds with different mechanisms of action: caffeoyl glycosides quercetin-3-*O*-(6-caffeoyl)-β-D-galactopyranoside (**47**), kaempferol-3-*O*-(6-caffeoyl)-β-D-galactopyranoside (**19**), kaempferol-3-*O*-(6-caffeoyl)-β-D-glucopyranoside (**17**), and hyperoside-6″-*O*-caffeoyl (**38**) actively inhibit α-glucosidase and can be used in the treatment of diabetes [37,53].

*S. media* and *S. humilis* are distinguished by a high level (up to 57 mg/g) of other polyphenolic compounds, such as catechins, in leaves and inflorescences [71]; for example, the concentration of catechins exceeds 30 mg/g (of dry matter) in *S. dahurica* and 18 mg/g in *S. salicifolia* [38]. Unlike other flavonoids, the catechins of spireas include mainly known compounds (Table 1).

It is worth mentioning the main types of biological activity of catechins. The active substances isolated from *S. hypericifolia* have antitumor activity. Aglycones (+)-catechin (**56**) and (−)-epicatechin (**62**) and their glycosides 7-α-L-rhamnopyranoside (+)-catechin (**59)**, 7-β-D-xylopyranoside (+)-catechin (**61**), and others have relatively low toxicity and exert anticancer action both on their own and in combination with irradiation in in vivo and in vitro experiments. The highest tolerated doses of flavans are 60–100 mg/kg (of body weight) in experimental animals. When rats with Pliss lymphosarcoma and mice with sarcoma 180 are treated with polyflavans, significant antitumor activity is seen. (+)-Catechin-7-α-L-rhamnopyranoside (**59**) when combined with radiation enhances the inhibition of tumor growth by 30% in rats with Pliss lymphosarcoma [56].

*Spiraea* species of the section *Spiraea* have pink inflorescences; accordingly, only in inflorescences of this section’s members, anthocyanins (1 mg/g) have been found [71]. All other species of *Spiraea* have white flowers and do not contain anthocyanins. It has been reported that spireas contain a family of procyanidins: cyanidin and procyanidins B1, B2, and C1 have been detected in flowering shoots of *S. salicifolia*, whereas procyanidins B1 and B2 (procyanidins) in the shoots of *S. hypericifolia* [32,52,72,73].

In studies on seasonal dynamics of phenolic compounds in spireas’ leaves, it has been noted that the profile of aglycones remains relatively constant during the growth season, with certain changes in the profile of flavonol glycosides. Because the studied sets of morphological characteristics do not fully describe the natural diversity of spireas, as early as 1993, T.W. Kim and Y.M. Lee proposed to study the natural and introduced populations of spireas growing in Korea, in terms of the traits related to the profile and levels of phenolic compounds [74]. The characterization of chromatographic profiles of phenolic compounds is also successfully used to clarify the taxonomy of *Spiraea* plants growing in Russia and Ukraine [46,48,49,73]. The investigation of intraspecific variability allows to identify the largest number of compounds and to distinguish among them major (constant) compounds and minor ones, i.e., those that are not present in all phases of plant vegetation or not present in all samples [59]. The determination of flavonoid profiles is especially relevant for the controversial species that are classified by most botanists as subspecies or varieties. For example, the present authors have uncovered significant differences in the profiles of phenolic compounds between *S. humilis* and *S. salicifolia*, which allows us to regard *S. humilis* as an independent species; in *S. sericea*, on the contrary, no signs of species independence have been found [48,75].

Analysis of the variability of the phenolic-compound profile and levels after the introduction of plants into a new environment has shown that after the introduction, there are changes in the profile of phenolic compounds, while there is a relatively stable pattern of phenolic compounds in the specimens growing in the wild. For instance, differences have been registered in high-performance liquid chromatography profiles of phenolic compounds between *S. betulifolia* subsp. *aemiliana* specimens from natural and introduced populations. Five new phenolic acids and one flavonol were found in the leaves of the introduced population. After *S. betulifolia* subsp. *aemiliana* were transferred from nature (Kunashir Island) to a novel environment (Novosibirsk), there was also a change in the levels of some phenolic compounds. The major phenolic compound is hyperoside (3.36–9.99 mg/g) in leaves from natural populations and quercetin (2.86–5.07 mg/g) in the introduced population [76]. Plant samples collected in introduced populations should be used in taxonomic studies with caution. The chemotaxonomy of spireas and other plants must be investigated using plenty of natural and introduced-population material, taking into account morphological data on the studied plants [49].

The presence of coumarins in studied spireas has been noted by many authors; it has also been established that the level of coumarins is usually higher in inflorescences than in leaves [71]. However, it has not yet been possible to isolate individual compounds of this class, except for a coumarin extracted from *S. salicifolia* shoots [52].

Phenolic acids are synthesized in shoots, leaves, and flowers of spireas (Table 2).

Among spireas, the profile of phenolic acids and their derivatives is most studied in *S. salicifolia*. In addition to the acids listed in the table, salicylic acid is present in the leaves of this species, whereas in flowering shoots, researchers have found 1-*O*-caffeoylglucose, 1-*O*-coumaroyl-β-D-glucopyranoside, 1-*O*-caffeoyl-β-D-glucopyranoside, 6-*O*-*cis*-*n*-coumaroyl-β-D-glucopyranoside, and 6-*O*-*trans*-*n*-coumaroyl-β-D-glucopyranoside [32,52,58,63,64]. In the *S. canescens* whole plant, other phenolic acid derivatives have been found: 6′-*O*-*p*-coumaroyl-α/β-D-glucopyranose, 6′-*O*-*p*-cinnamoyl-α/β-D-glucopyranose, and 6′-*O*-(4-methoxy-*trans*-cinnamoyl)-α/β-D-glucopyranoside [29]. Several phenolcarboxylic-acid derivatives have been identified in the branches of *S. formosana*: nonadecyl ferulate, methyl ferulate, ethyl ferulate, ethyl-*p*-hydroxy-*trans*-cinnamate, methyl vanillate, and 3-*O*-β-D-glucoside-*p*-vanillic acid [34], and in *S. prunifolia* leaves, researchers have found 1-*O*-cinnamoyl-β-D-glucopyranose, 1-*O*-*p*-coumaroyl-β-D-glucopyranose, 1,2-*O*-dicaffeoyl-β-D-glucopyranose, 1,6-*O*-dicaffeoyl-β-D-glucopyranose, and 1-*O*-caffeoyl-β-D-glucopyranose [31]. In roots of *S. prunifolia* var. *simpliciflora*, investigators have detected *p*-hydroxycinnamic acid methyl ester [60,81]. New phenol glycosides have been identified in spireas. For instance, from twigs of *S. prunifolia* var. *simpliciflora*, investigators have isolated 1-O-(*E*)-caffeoyl-2-*O*-p-(*E*)-coumaroyl-β-D-glucopyranoside [82], and from the *S. canescens* whole plant, 6″-*O*-*trans*-*p*-coumaroyl-(4-hydroxybenzoyl)-*β*-*D*-glucopyranoside [29].

The greatest amount of phenolcarboxylic acids accumulates in leaves and flowers; it is also reported that more flavonoids and phenolcarboxylic acids accumulate in (nonlignified) stems of the current year than in lignified stems (second and third years of life) [37,40]. The research on the dynamics of the accumulation of flavonoids and phenolcarboxylic acids among growth season phases has shown that their level is the highest during the flowering period, and it has been demonstrated (using plants growing in Irkutsk Oblast and Buryatia as an example) that the dependence of phenolic compounds’ levels on the sites of collection results in only insignificant fluctuations [52].

From a leaf extract of *S. prunifolia* var. *simpliciflora*, five caffeoyl hemiterpene glycosides have been isolated: 4′-(6-*O*-caffeoyl-β-D-glucopyranosyl)-2′-methylbutyric acid, 1,4′-dicaffeoyl-6-tuliposide B, 1-caffeoyl-6-tuliposide A, 1,2-dicaffeoyl-6-tuliposide A, and its structural isomer, namely, 1-*O*-caffeoyl-6-*O*-(4′-caffeoyl-2′-methylene-butyroyl)-β-D-glucopyranoside [60]. The last four compounds (if we disregard the caffeoyl moiety) are based on a specific glucoside called 6-tuliposide A [6-O-(2′-methylene-4′-hydroxy-butyroyl)-α/β-D-glucopyranose], previously found in the leaves of *S. salicifolia* and *S. thunbergii*. Besides, tulipalin A, more commonly known as α-methylene-γ-butyrolactone, has been detected in these species [37,83,84]. Chemical group α-methylene-γ-butyrolactone (especially its open-chain derivatives) is regarded as an active center of some compounds and as the reason for the insecticidal and other activities of these substances [85]. For instance, recently, a family of new natural cinnamoyl glucosides—containing such a group and inhibiting plant growth—has been isolated from several *Spiraea* species [86,87]. Their isolation has been carried out by various modern methods and, in most studies, has been accompanied by testing of biological activity. For example, a new diacetyl carbohydrate called 6-*O*-(3′,4′-dihydroxy-2′-methylenbutyryl)-1-*O*-*trans*-cinnamoyl-β-D-glucopyranose was isolated from the leaves of *S. alpina* collected in Sichuan province (China) [85,88]. From flowering shoots of *S. salicifolia* collected in the Tunkinsky district (Buryatia, Russia), investigators have isolated 1-*O*-*trans*-cinnamoyl-6-*O*-(4″-hydroxy-2″-methylenbutyryl)-β-D-glucopyranose and 1-*O*-*cis*-cinnamoyl-6-*O*-(2′-methylen-4′-hydroxybutyryl)-β-D-glucopyranose [32]. In the *S. canescens* whole plant, other new compounds have been found: (6′-*O*-*trans*-cinnamoyl)-(2-hydroxymethyl-4-hydroxy-butenyl-β-D-glucopyranoside, 2-(*trans*-cinnamoyloxy-methyl)-1-butene-4-*O*-β-D-glucopyranoside, and *O*-*trans*-*p*-coumaroyl-(4-hydroxybenzoyl)-β-D-glucopyranoside [29]. From the leaves of *S. prunifolia* var. *simpliciflora*, xylosides [i.e., hexyl-β-primeveroside and (*Z*)-hex-3-enyl-β-primeveroside] [31] as well as a new compound [1-*O*-*cis*-*p*-coumaroyl-6-*O*-(4-hydroxy-2-methylenebutanoyl)-β-D-glucopyranose] [62] have been isolated. In flowers of *S. salicifolia*, researchers have identified 1,6-dihydroxy-2-methylanthraquinone [40].

In recent years, valuable biological properties were demonstrated for some hemiterpene glycosides. S.H. Park et al. [60] have found that substances such as hemiterpene glycosides (1-caffeoyl-6-tuliposide A, 1,40-dicaffeoyl-6-tuliposide B, and 1,2-dicaffeoyl-6-tuliposide A), isolated from *S. prunifolia* leaves have anti-inflammatory properties. These scientists have also determined that the structure of the hemiterpene moiety in the isolated compounds has a strong effect on cell permeability and inhibits nitric oxide production [60]. J. Lee et al. [89] have demonstrated antimicrobial activity against *Escherichia coli* in butyrolactone (S-(-)-tulipalin B) isolated from *S. thunbergii*. The major fungitoxic constituent of *S. alpina* is a new diacylated sugar structurally identified as 6-*O*-(3,4′-dihydroxy-2′-methylenbutyryl)-1-*O*-*trans*-cinnamoyl-β-D-glucopyranose. This compound at 0.1 mg/mL can inhibit the growth of *Rhizoctonia solani* and *Exserohilum turcicum* by 87.6% and 63.2%, respectively [85]. Caffeoyl-6-tuliposide A and 1-*O*-*cis*-*p*-coumaroyl-6-*O*-(4-hydroxy-2-methylenebutanoyl)-β-D-glucopyranose from the twigs of *S. prunifolia* var. *simpliciflora* are relatively potent stimulants of nerve growth factor secretion by C6 cells and therefore can be considered neuroprotective substances [62]. Hemiterpene glycoside 1-caffeoyl-6-tuliposide A, bioactive α-hydroxylated lactone (–)-nortrachelogenin, and lignans (+)-fraxiresinol and 4-*O*-β-D-glucopyranoside from *S. prunifolia* var. *simpliciflora* are strongly toxic to non–small cell lung adenocarcinoma cells (A549), ovarian cancer cells (SK-OV-3 and SK-OV-3), kidney carcinoma cells (A498), and colon cancer cells (HCT15). Researchers also report that the presence of the 4-hydroxy-2-methylene-butyrate residue at position C-6″ in the glucose unit is important for the toxicity to cell lines A549, SK-OV-3, and A498 [82].

Among the active substances isolated from *Spiraea* plants, there are also compounds with high antioxidant activity. Hemiterpene glycosides and dicaffeoyl glycosides from *S. prunifolia* leaves have a greater DPPH radical-scavenging activity than L-ascorbic acid does (IC_50_ = 24.99 ± 0.35 μM), which served as a positive control. 40-Hydroxy-20-methyl-butyrate with a substituted hemiterpene moiety showed the highest activity in this assay [60]. It was therefore hypothesized that the carboxyl group of the butyrate stabilizes the free radicals by acting as an electron-donating group. The hemiterpene moiety proved to be important for free-radical-scavenging activities. Superoxide scavenging was measured too to evaluate the antioxidant activities of the compounds isolated from the leaves of *S. prunifolia*. Compounds bearing two caffeoyl groups exhibited better antioxidant actions than those containing only one. These results suggest that the number of caffeoyl groups in these isolated compounds is important for the elimination of superoxide [60].

In a methanolic extract from the leaves and branches of *S. prunifolia* var. *simpliciflora*, researchers have found isosalicin, crenatin (has a neuroprotective effect), vanilloside, (4-hydroxy-3,5-dimethoxyphenyl)-methyl-β-D-glucopyranoside, hydrangeifolin I, tachioside, isotachioside, koaburside, 1-hydroxy-3,4,5-trimethoxyphenyl-1-*O*-[6′-*O*-(4″-carboxy-1″,3″,5″-trihydroxy)phenyl]-β-D-glucopyranoside, eugenol 4-*O-β*-primeveroside, vanillin, syringaldehyde, and 2-(2-hydroxy-5-methoxyphenyl) ethanol [31,62,82,90,91]. In an ethanolic extract of *S. formosana* branches, scientists have found the phenolic lactone agrimonolide and a benzene derivative: *p*-hydroxybenzaldehyde [34], whereas in a methanolic extract of *S. blumei*, 6-hydroxyeugenol has been identified, which prevents or slows the progression of atherosclerosis [92].

Some spireas have been reported to contain known compounds from the class of stilbenes. For instance, from an ethanolic extract of *S. formosana* branches, researchers have isolated 3-*O*-β-D-glucoside-5,4′-dihydroxystilbene and 3,5,4′-trihydroxystilbene [34], and resveratrol has been found in the leaves of *S. trilobata* [57].

**Figure 2:**
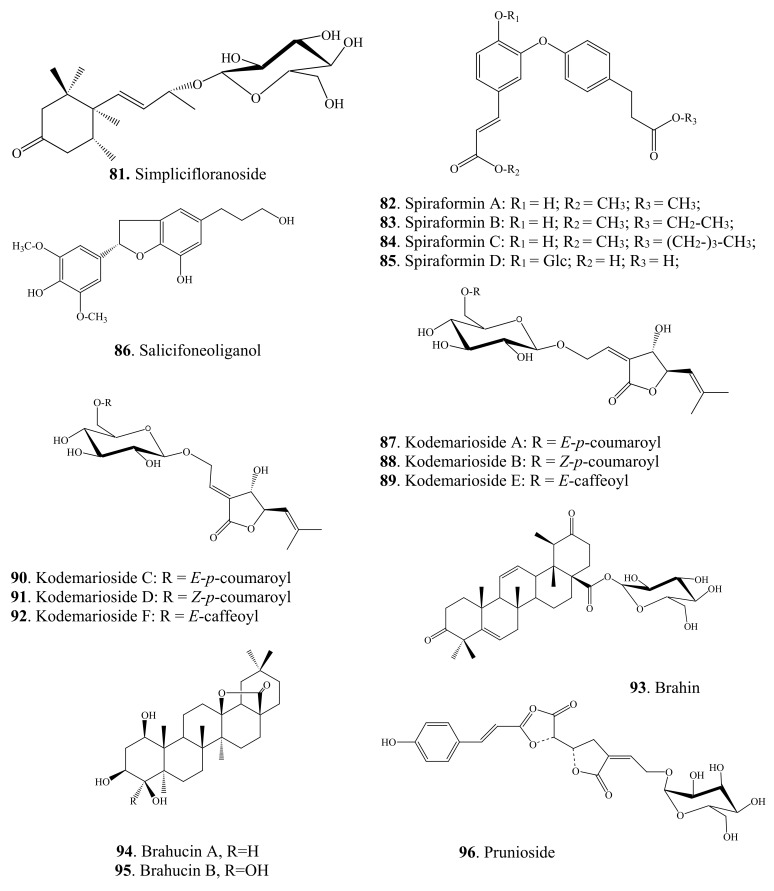
Other new secondary metabolites found in spireas.

Lignans are another family of polyphenolic compounds isolated from spireas. Most often in spireas, investigators find (+)-lyoniresinol, (±)-syringaresinol, (+)-isolariciresinol, (+)-africanal, and (+)-cycloolivil. In addition to the above lignans, derivatives of isolariciresinol have been found in *S. japonica* var. *ovalifolia*: isolariciresinol-9-*O*-β-D-xylopyranoside and (4),5-methoxy-isolariciresinol-9-*O*-β-D-xylopyranoside [93]. In *S. pubescens*, researchers have found 5-methoxy-(+)-isolariciresinol, (+)-lyoniresinol-9-*O*-β-D-xylopyranoside, (–)-lyoniresinol-9-*O*-β-D-xylopyranoside, pinoresinol-4′-*O*-glucopyranoside, 8-hydroxypinoresinol-4′-*O*-β-D-glucopyranoside, and (–)-nortracheloside [31,94]. In the *S. canescens* whole plant, they have identified (+)-lyoniresinol-3a-*O*-β-D-glucopyranoside and (+)-isolariciresinol-3a-*O*-β-D-glucopyranoside [29]. Phytochemical investigation of a methanolic extract from the twigs of *S. prunifolia* var. *simpliciflora* by column chromatography has led to the isolation of 10 known lignans: (–)-nortrachelogenin, lariciresinol, (–)-olivil, (–)-berchemol, (+)-1-hydroxypinoresinol, (+)-fraxiresinol, (+)-1-hydroxypinoresinol 1-*O*-β-D-glucopyranoside, (–)-secoisolariciresinol, (+)-9-*O*-β-D-glucopyranosyl lyoniresinol, and (+)-9-*O*-β-D-glucopyranosyl isolariciresinol as well as 7*R*,8*S*-5-methoxydihydrodehydroconiferyl alcohol, 7*R*,8*S*-dihydrodehydrodiconiferyl alcohol, and dihydrodehydrodiconiferyl alcohol 4-*O*-β-D-glucopyranoside [82]. In addition to naturally occurring fraxiresinol, 8-hydroxy-7′-epipinoresinol, and 8-hydroxypinoresinol, other compounds have been isolated from an ethanolic extract of *S. salicifolia*, among which, a previously uncharacterized lignan has been found, i.e., 7*S*,8*R*-3,5-dimethoxy-4′,7-epoxy-8,5′-neolignan-3′,4,9,9′-tetraol, named salicifoneoliganol (**86**, Figure 2) [36]. In an analysis of the underground part of the same species, a new neolignan glycoside [9-O-α-L-rhamnopyranoside (7S,8R)-3,5-dimethoxy-7,4′-epoxy-8,5′-neolingan-4,9,3′,9′-tetraol] was isolated whose structure was confirmed by spectroscopic data. Two known neolignane glycosides have been found too: (7*S*,8*R*)-3-methoxy-4′,7-epoxy-8,5′-neolignan-3′,4,9,9′-tetraol 9-*O*-*α*-L-rhamnopyranoside and (7*R*,8*S*)-dihydrodehydrodiconiferyl alcohol 9′-*O*-*β*-D-glucopyranoside [95]. The latter compounds have been previously found only in *Juniperus communis* L. var. *depressa* Pursh. [96]; these compounds were isolated from spireas for the first time. From an ethanolic extract of *S. formosana* branches, in addition to the known compound (–)-isolariciresinol-3a-O-β-D-glucopyranoside, four neolignans named spiraformins A–D (**82**–**85**, Figure 2) have been isolated for the first time; their structures have been characterized by NMR spectroscopy [34].

The roots of spireas are known as multifunctional plant raw material. Many valuable applications of these species were proved in scientific studies recently. Neolignan glycosides from the roots of *S. salicifolia* have been subjected to an assay of inhibition of the production of proinflammatory cytokine interleukin-6 in lipopolysaccharide (LPS)-stimulated RAW 264.7 cells, and all the compounds have shown anti-inflammatory effects [95]. Although naturally occurring prunioside A from *S. prunifolia* var. *simpliciflora* roots showed no inhibitory effect, its derivatives have inhibitory effects on nitric oxide production in murine macrophagelike RAW 264.7 cells stimulated with LPS and interferon-γ and may be useful for the treatment of inflammatory diseases [97,98,99,100].

### 3.2. Biotechnological Studies on Plants of the Genus Spiraea for Assessing the Ability to Accumulate Phenolic Compounds in In Vitro Culture

Currently, methods for the culturing of plant cells, tissues, and organs are among the tools for basic research into the processes of plant growth and development and have practical importance [101]. *In vitro* culture is a popular plant propagation technology for the production of valuable secondary metabolites [102,103]. Despite the strong interest in *Spiraea* members as ornamental plants and sources of secondary metabolites, information on the microclonal propagation of spireas is limited. There are only a few reports on in vitro culture of spireas (about *S. bumalda* “Anthony Waterer” [104], *S. × vanhouttei* [105], *S. nipponica* [106], *S. cana* [107], and *S. crenata* [108]). A callus of *S.* × *vanhouttei* has been successfully initiated on a sucrose medium [109].

The level of phenolic compounds in representatives of the genus *Spiraea* in in vitro culture has been estimated only in one species [110]. Micropropagation protocols for *S. betulifolia* subsp. *aemiliana* were developed for the first time. This plant from two natural populations—on Sakhalin and Kunashir Islands—was introduced into in vitro culture to assess the ability to accumulate phenolic compounds in in vitro microshoots as compared with intact plants. Active adventitious shoot formation for the Sakhalin plants was implemented on the Murashige–Skoog (MS) nutrient medium supplemented with 0.1 μM 6-benzylaminopurine (18.8 ± 6.0 shoots per explant; 90% regeneration frequency) and for Kunashir plants on MS containing 5.0 μM 6-benzylaminopurine and 1.0 μM 1-naphthalene acetic acid (28.8 ± 3.6 and 91%, respectively). Indole-3-butyric acid was found to be effective for the in vitro rooting; the best result was obtained on ½ MS supplemented with 0.1 μM indole-3-butyric acid. By high-performance liquid chromatography, it was determined that the profile of phenolic compounds was more diverse in intact plants than in the plants from in vitro culture: 26 versus 13–21 phenolic compounds, respectively. Concentrations of chlorogenic acid, kaempferol, and “phenolic acid 4” were 1.5–2.0-fold higher in the in vitro plants (depending on the cultivation stage) compared to the intact plants, while concentrations of hyperoside, astragalin, and quercetin were higher in the intact plants. Research data indicate feasibility of the use of *S. betulifolia* subsp. *aemiliana* plants from in vitro culture as a valuable alternative source of phenol carboxylic acids and flavonoids as well as potential usefulness of further investigation into the accumulation of secondary phenolic compounds in this subspecies in vitro [110].

### 3.3. Nitrogen Compounds

In one of the first chemical studies regarding nitrogen-containing substances in spireas, isoamylamine was found in *S. media* flowers [111]. Subsequent studies (mainly on *S. japonica* and its varieties) have led to the isolation and identification of more than 80 alkaloids belonging to the atisine, hetisine, and atisane families. Chemotaxonomy has been proposed based on the structures described for diterpene alkaloids. It is believed that the Southwest of China is the center of modern differentiation, probably the original center of the *S. japonica* complex. The features of these alkaloids, as well as *S. japonica* chemotaxonomy on the basis of the isolated substances are reviewed in more detail by X. Hao et al. [25]. After the publication of that review, from the aerial part of *S. japonica* var. *acuminata* Franch, Y. Ma et al. [112] have isolated 11 diterpene alkaloids of atisine and atisane types (spiramine A, B, C, C-2, D, F, P, and Z), spiramilactone B and G, and spiramine C, previously found in other varieties of *S. japonica*. The atisine type also includes spiramilactams A and B isolated from the aerial part of *S. japonica* var. *ovalifolia* [113], whereas the atisane type includes new compounds spiramilactone E and spiratisanins A–C from the aerial part of *S. japonica* var. *acuta* [114,115] and spiraeosides A and B from *S. japonica* var. *ovalifolia* [116]. The hetisine type includes spiraqine, 6-hydroxyspiraqine, spiradines A–C, and spirasines II, III, V, and VI from the aerial part of *S. japonica* var. *fortunei* [117,118]. In the aerial part of *S. japonica* var. *ovalifolia*, in addition to diterpene alkaloids of the atisine type, new compounds from the class of diterpene glucosides of the atisine type have been found for the first time, which are extremely rare in plants [116]. Studies on alkaloids of the *S. japonica* complex, for example, have confirmed the validity of the subdivision of spireas’ alkaloids into three types and have shown that the *S. japonica* complex is a special and relatively independent group within spireas. A hypothesis has been advanced that the complicated *S. japonica* complex has originated in the Southwest of China and has an evolutionary tendency from west to east; this hypothesis has been proven successfully [117]. 

In addition to *S. japonica*, alkaloids have been detected in some other spireas: in *S. salicifolia*, alkaloid spiradine F has been identified, whereas in the leaves of *S. koreana*, spirajine has been found [119]; in branches of *S. formosana*, spiraeaine A has been registered [34,120], and in the roots of *S. fritschiana* var. *parviflora*, spirafines II and III and spiradine D have been detected [121]. Screening of *Spiraea* species for alkaloids revealed that extracts of *S. crenata*, *S. nipponica*, *S. × vanhouttei*, *S.* × *billardii*, and *S. media* do not contain alkaloids, and these compounds have been detected only in the roots of *S. chamaedryfolia* [122]. Nevertheless, in that study, it was not possible to isolate alkaloids from this species either, because of the low stability of these compounds; at the same time, it was determined that alkaloids accumulate in the secondary cortex and secondary xylem of the *S. chamaedryfolia* root, while alkaloids are absent in the core [122]. A nitrogen-containing compound called aurantiamide acetate has been found in an ethanolic extract of *S. formosana* branches [34]. Some alkaloids of spireas have been successfully synthesized artificially [123].

Research conducted in recent years focused on biological activity of alkaloids from *S. japonica* extracts. Some alkaloids from spireas have been shown to possess substantial bioactivity, including anti-inflammatory, anti-platelet aggregation, and neuroprotective effects [25]. Spiramine Q, an atisine-type diterpene alkaloid from *S. japonica* var. *incisa*, has been shown in vitro and ex vivo to decrease mouse mortality caused by intravenous injection of arachidonic acid [124]; in the study just cited, spiramine Q was more active than aspirin. Spiramine T, an atisine-type diterpene alkaloid from *S. japonica* var. *acuta*, exerts protective effects against cerebral ischemia-reperfusion injury in gerbils, and its mechanism of action may be related to the inhibition of calcium overload, antiperoxidation effects, and modulation of an endogenous antioxidant [125]. Spiramine C1 from *S. japonica* var. *acuta* concentration-dependently inhibits the platelet aggregation induced by platelet-activating factor, adenosine-5′-diphosphate, and arachidonic acid with IC_50_ of 30.5, 56.8, and 29.9 μM, respectively; these data are suggestive of a nonselective anti-platelet aggregation action. The inhibitory influence of spiramine C1 on arachidonic acid is reported to be as potent as that of aspirin [126]. Spiramine N-6 inhibits platelet aggregation *in vitro* and *in vivo*, decreases a serotonin release from platelets, and suppresses the binding of activated platelets to neutrophils [127]. Compounds isolated from the *S. japonica* species complex possess an anti-tobacco mosaic virus activity. Moreover, diterpene alkaloids with an imine group at position C-20 and an acetyl group at position C-6 have more potent anti-tobacco mosaic virus activity (protective effect) than other compounds do [112]. Atisine-type diterpene alkaloids from the ethanolic extract of *S. japonica* at a concentration of 100 μg/mL can inhibit the infection by tobacco mosaic virus and show in vivo curative properties, with the cure rate of 55.4% for hsp-X26, 53.2% for hsp-X31, 58.1% for hsp-X35, and 60.3% for hsp-X40, which is better (*p* < 0.05) than that of a positive control, ningnanmycin (55.2%). The diterpene compound hsp-X40 showed the best inhibitory activity (78.1%). The anti-tobacco mosaic virus activity of atisine-type diterpene alkaloids from *S. japonica* is related to downregulation of the tobacco mosaic virus coat protein [128]. Chinese scientists [129,130] have revealed an anticancer activity of diterpene alkaloids from *S. japonica*. A spiramine derivative (with a β-unsaturated ketone group) isolated from *Spiraea* species, is a novel anticancer agent capable of inducing apoptosis in cancer cells.

### 3.4. Terpenoids

Terpenoids of the above-ground organs of spireas are represented by known compounds. For example, β-amyrin was found in *S. formosana*, *S. salicifolia*, and *S. pubescens*; lupeol in *S. pubescens* and *S. salicifolia*; and oleanolic acid in *S. brahuica* and *S. salicifolia*; besides, in *S. brahuica*, researchers found 3-*O*-(β-D-glucopyranosyl)-oleanolic acid and betulinic acid; an extract of *S. japonica* var. *ovalifolia* contains 18-hydroursolic acid, the roots of *S. prunifolia* var. *simpliciflora* contain ursolic acid and tormentic acid, *S. pubescens* contains lupeone, fridelin, betulin, and uvaol, whereas *S. cantonensis* is a source of 3-*epi*-betulinic acid [77,93,131,132,133,134]. The diterpenoids of *S. salicifolia* are represented by dehydroabietic acid and its methyl ester; as for triterpene compounds, researchers have identified ursolic acid and glycyrrhizinic acid as well as 3β-acetylursolic acid methyl ester and 3β-acetyloleanolic acid methyl ester [36,52]. From an ethanolic extract of *S. formosana* branches and *S. thunbergii* stalks, scientists have isolated glutinol, taraxerol, and bakuchiol, and from *S. tosaensis* stalks, they have isolated glutinone [34,131]. From a methanolic extract of the above-ground part of *S. brahuica*, investigators have isolated diterpenes: marrubiin, 19-acetylmarrubenol, and 6-acetylmarruenol [135], and from the twigs of *S. prunifolia* var. *simpliciflora*, lupine-type triterpenoids: taraxerol, β-amyrin, germanicol, and germanicone as well as methyl 3-*O*-acetylbetulinate and 3-*O*-acetylpomolic acid methyl ester [62,81]. Kodemariosides A–F (**87**–**92**, Figure 2) are among the new terpene compounds found in flowers and leaves of *S. cantoniensis* and represent new monoterpene acylglucosides [35]. Aside from these compounds, in the leaves of this species, investigators have found 3,7-dimethyl-2(*E*),6-octadien-1-*O*-β-D-glucoside, 3,7-dimethyl-3(*E*),6-octadien-5-one-1-*O*-β-D-glucoside, and 3,7-dimethyl-3(*Z*),6-octadien-5-one-1-*O*-β-D-glucoside [136]. These compounds contain a unique cross-conjugated dienone system similar to that seen in *cis*-atlantone and its *trans*-isomer isolated from *Cedrus* sp. [137]. Three new compounds have been identified in *S. brahuica*: brahin (**93**, Figure 2) and brahucins A and B (**94**, **95**, Figure 2), the first one is an ursen-type compound, and the other two belong to the olean type [77,79]. A new terpene glucoside—prunioside A (**96**, Figure 2)—has been isolated from a methanolic extract of *S. prunifolia* var. *simpliciflora* roots [97,100].

The most recent studies on pharmacological properties of spirea extracts revealed a beneficial property of terpenoids: brahin (**93**, Figure 2) showed a moderate inhibitory activity against lipoxygenase with IC_50_ of 56.75 µM, as compared with IC_50_ of 8.01 µM observed for baicalein serving as a positive control [79]. Compounds from *S. prunifolia* var. *simpliciflora* (β-amyrin, methyl 3-*O*-acetylbetulinate, and β-sitosterol) show moderate toxicity to some of the four tested human cancer cell lines (A549, SK-OV-3, SK-MEL-2, and BT549), with IC_50_ ranging from 23.59 to 29.71 μM [62]. Furthermore, such compounds as methyl 3-*O*-acetylbetulinate and vanillin from the twigs of *S. prunifolia* var. *simpliciflora* cause mild inhibition of nitric oxide production in LPS-stimulated murine microglial BV-2 cells [62]. 

Megastigmanes belong to the class of terpenes, a subclass of sesquiterpenes, but due to their specific C13 skeleton, they got a separate name: isonorterpenoids or megastigmanes. Megastigmanes are found in many plants, such as *Cucumis sativus* and *Juniperus communis* [138]. From the aerial part of *S. prunifolia* var. *simpliciflora*, a new megastigmane glycoside named simplicifloranoside (**81,** Figure 2) has been isolated [31].

In most species (*S. humilis*, *S. beauverdiana*, *S. salicifolia*, *S. latifolia*, and *S. media*) from the living collection of the Botanical Garden at the Institute of Biology, Komi Scientific Center (the Ural Branch of the Russian Academy of Sciences) and from the natural population, the highest level of saponin-containing extractive substances was found in inflorescences (1.8% to 8.1% of dry weight), while the highest level of these substances was found in inflorescences of *S. corymbosa* (8.1%) and in leaves and inflorescences of *S. betulifolia* (7.1% and 7.2%, respectively) [139]. In leaves of all the tested species, except for *S. betulifolia*, saponin-containing substances were found [0.8% (*S. salicifolia*) to 3.0% (*S. media*)]. By thin-layer chromatography and high-performance liquid chromatography, among the obtained saponins, five to six individual compounds have been identified, including diosgenin: the genin of most of spirostanol glycosides [139].

Among known steroids from the branches and leaves of *S. pubescens, S. formosana*, *S. brahuica*, *S. mongolica*, and *S. prunifolia* var. *simpliciflora,* there are β-sitosterol and stigmasterol; besides, in *S. formosana,* there are β-sitosteryl glucoside and β-sitosterone; in *S. japonica* var. *ovalifolia*, daucosterol; and in *S. pubescens*, stigmastanol and stigmast-4-en-3-one [27,34,77,93,94,120,140]. In the roots and twigs of *S. prunifolia* var. *simpliciflora*, sterols campesterol and β-sitosterol-3-*O*-β-D-glucopyranoside have been found [90,133].

### 3.5. Polysaccharides

Spireas are distinguished by a high concentration (up to 20.7%) of polysaccharide complexes (more than 69% in flowers), which include mono- and disaccharides and uronic acids [71,141].

### 3.6. Higher Fatty Acids

The fatty acid composition of *S. salicifolia* and *S. hypericifolia* shoots has been studied and is now known to include palmitic, stearic, linoleic, and linolenic acids; besides, in *S. hypericifolia*, investigators have found myristic, pentadecanoic, and palmitoleic acids [142], whereas in *S. salicifolia*, they have detected arachidic, behenic, tricosanoic, or lignoceric acid as well as higher aliphatic carbohydrates: hexylheptadecane, octadecane, pentacosane, eicosane, heneicosane, docosane, tricosane, and tetracosane [52]. Among the lipids of *S. media* leaves and inflorescences, palmitic and linolenic acids are predominant. Saturated fatty acids dominate among lipids in leaves, significantly exceeding the level of unsaturated fatty acids. There are rather high concentrations of such rare unsaturated acids as vaccenic and gadoleic and saturated acids: behenic and lignoceric [143]. Spiraeamide, a novel sphingolipid, has been found in a methanolic extract of the *S. brahuica* aerial part [135].

### 3.7. Organic Acids

The profile of organic acids in the flowers and leaves of *S. salicifolia* is known: researchers have found acetimidic, butyric, succinic, fumaric, and 3-ketoglutaric acids [52,141]. The amino acid composition of the above-ground and underground parts of *S. hypericifolia* has been researched in detail, and the levels of macro- and microelements in *S. hypericifolia* were determined too. The major amino acids in *S. hypericifolia* are glutamate, aspartate, and alanine [142]. The accumulation of ascorbic acid has also been studied in leaves of 22 *Spiraea* species introduced into the Belgorod region. For most species of *Spiraea*, there is a tendency toward a decrease in the concentration of ascorbic acid during the growth season. Leaves of *S.* × *rubella* and *S. canescens* collected in the first month of summer and leaves of *S. ussuriensis* collected in the last month of summer have been proposed for therapeutic use. It was demonstrated that *S.* × *rubella* contains the highest concentration of ascorbic acid among the studied species [144]. Sanleng acid has been isolated from *S. japonica* var. *ovalifolia* [93].

### 3.8. Elemental Composition

In the leaves and flowers of *S. salicifolia* from Eastern Siberia, nine macro- and 54 micro- and ultramicroelements have been detected. Among macronutrients, there is a high concentration of potassium, calcium, magnesium, and phosphorus; among trace elements, high concentrations of manganese, zinc, and copper are present. Flowers accumulate more iron and copper than leaves do [52,145]. In the aerial and underground organs of *S. hypericifolia,* there are 11 macro- and microelements according to multielement atomic emission spectral analysis, and major elements among them are Ca, K, and Mg [142]. The levels of trace elements have been studied in *S. dasyantha* too [146].

### 3.9. Essential Oils

Spireas are not typical aromatic plants; therefore, there is very little information about essential-oil composition of *Spiraea* species. The profile of essential oils has been researched in detail in three species: *S. hypericifolia*, *S. alpina*, and *S. mongolica* [26,27,28]. Essential oils have been isolated from the aerial parts (leaves and flowers) of *S. hypericifolia* collected in Northern Kazakhstan [28]. Aliphatic hydrocarbons (alkanes) (40.6–53.2%), aldehydes (8.4–17.4%), diterpenoids (9.1–16.7%), and ketones (6.2–8.7%) were the major compounds. *n*-Heneicosane (17.4–34.1%) and *n*-tricosane (14.3–19.5%) were found to be the main constituents of the essential oil from *S. hypericifolia*. Practically important compounds, such as α-methylene-γ-butyrolactone (0.8–2.8%), benzyl cyanide (0.7–1.1%), β-damascenone (1.2–2.9%), (*E*,*E*)-4,8,12-trimethyl-1,3,7,11-tridecatetraene (1.8–2.7%), β-ionone (0.5–1.8%), and others have been detected only in small amounts [28]. Carboxylic acids, monoterpenes, and diterpenes are abundant in the leaf oil from *S. alpina*, representing 49.95% [26]. The major compounds were linoleic acid ethyl ester, palmitic, and cinnamic acids, their esters, and other derivatives, as well as *trans*-phytol and linalool (some of them are probably artefacts of the isolation procedure). Additionally, sesquiterpenes and alcohols (hexahydrofarnesyl acetone, α-terpineol, nerolidol, β-ionone, and others) were detected as minor constituents [26]. The essential oils isolated from leafy shoots of *S. mongolica* are mostly based on ethyl palmitate (38.6%) and ethyl linolelaidate (23.5%), and other major compounds are botulin, lupine-3,20-diol, 1-β-hydroxyl-6,9-dien-8-oxoeremophil-11-nor-11-ketone, 3-(4-methoxyphenyl) propanal, stigmasterol, and β-sitosterol [27]. Overall, it has been documented that the essential oils in the studied *Spiraea* species are based on fatty and carboxylic acids and their derivatives, while traditional essential oils mostly contain terpenoids [147]. Microscopic examination of the aerial organs of *S. salicifolia* has not revealed any excretory organs (e.g., glands, glandular hairs, receptacles, or tubules) producing essential oils, but mono- and sesquiterpenes have been found in the leaves [(E)-ocimenone, isopulegone, and longipinane] and flowers (D-verbenone, farnesane, and hexahydroxyfarnesyl acetone). It has been demonstrated that components of the essential oil of *S. salicifolia* are formed in parenchymal cells and are dissolved in enchylema [52].

The essential oils of spireas have been tested for various types of activity. Antifungal in vitro activity has been documented for *S. alpina* essential oil [26]. In particular, in one study, this essential oil at a concentration of 10 μg/mL suppressed the growth of *Ralstonia solanacearum*, and at a higher concentration (up to 125 μg/mL), suppressed the growth of *R. solani*, *Fusarium graminearum*, and *E. turcicum* [26]. The essential oil from the leaves and branches of *S. mongolica* neutralizes free radicals of DPPH (IC_50_ = 900 μg/mL); on the other hand, this essential oil is reported to be inactive against *Bacillus subtilis*, *Aspergillus flavus*, and *Candida albicans* [27]. Nonetheless, its individual components such as 1β-hydroxy-6,9-diene-8-oxoeremophil-11-nor-11-ketone and 3-(4-methoxyphenyl)-propanal possess antibacterial and antifungal activities against the above microbes [27].

## 4. Biological Activity of Extracts and Their Individual Fractions from Plants of the Genus *Spiraea*

Spireas have been repeatedly studied worldwide not only regarding their chemical composition but also on the subject of various useful properties.

### 4.1. Anti-Inflammatory Activity

The attention of scientists from various countries is attracted by the anti-inflammatory activity of spireas (Table 3). Young leaves, fruits, and roots of *S. prunifolia* var. *simpliciflora* are used in Oriental medicine to treat pyretic and emetic diseases. A fever is induced as part of the acute phase response: a generalized reaction to infection and inflammation [22,97]. Anti-inflammatory effects of extracts from *S. prunifolia* var. *simpliciflora* have been studied repeatedly in vitro and in vivo. A methanolic extract from the roots of this plant is reported to significantly inhibit the formation of nitric oxide and superoxide in RAW 264.7 cells. This work showed low cytotoxicity of the extract from the plant’s roots: the methanolic extract did not affect the viability of RAW 264.7 cells even at 200 μg/mL. Overall, the antipyretic effects of the methanolic extract from the roots of *S. prunifolia* var. *simpliciflora* may be explained by direct inhibition of nitric oxide and a reduction in superoxide production [22]. An aqueous extract of *S. prunifolia* var. *simpliciflora* roots strongly suppresses the production of nitric oxide during an LPS-induced inflammatory response, without cytotoxicity. Furthermore, the extract decreases H_2_O_2_ cytotoxicity by enhancing cell viability and significantly reduces the intracellular level of reactive oxygen species. The cytotoxicity of the aqueous extract has been tested. The extract is reported to be nontoxic to RAW 264.7 cells at 250 μg/mL [80]. Lee et al. [24] have demonstrated that a methanolic extract of *S. prunifolia* var. *simpliciflora* leaves has a good potential for the treatment of acute lung injury. The extract of the leaves decreased the number of inflammatory cells and the levels of tumor necrosis factor, interleukin (IL)-1β, and IL-6 in bronchoalveolar lavage fluid and suppressed inflammatory-cell infiltration in lung tissue. The extract effectively inhibited airway inflammation and reactive oxygen species-mediated oxidative stress; this effect is closely related to its ability to induce activation of nuclear factor erythroid 2-ƒrelated factor and to inhibit the phosphorylation of mitogen-activated protein kinases and subunit p65 of nuclear factor κB. A methanolic extract from *S. prunifolia* leaves has also been shown to be nontoxic at 100 μg/mL [24]. 

Choi et al. [148] have examined the inhibitory effects of a methanolic extract of *S. fritschiana* on LPS-induced nitric oxide production in RAW 264.7 cells. The nitric oxide inhibition rate was 90% at 200 µg/mL methanolic extract. At the same concentration, the expression of proinflammatory genes, such as inducible nitric oxide synthase and cyclooxygenase, decreased too. The extract at 10–200 μg/mL does not affect cell viability [148].

The anti-inflammatory activity of *S. media* and *S. salicifolia* was confirmed in an in vivo experiment. A 50% aqueous-ethanolic extract from *S. media* leaves when administered intragastrically at a dose of 100 mg/kg had an anti-inflammatory effect at all stages of the inflammatory process. In rats, the extract reduced the degree of tissue alteration and enhanced the recovery of physiological processes at the site of inflammation caused by subcutaneous injection of 9% acetic acid into the back with simultaneous administration of a dextran solution. The area of tissue damage on days 9 and 29 was smaller by 14.43% and 25.71%, respectively. In this context, chamomile infusion had a weaker effect on the alterative phase of inflammation [23]. V.M. Mirovich and colleagues [149] have developed a method for obtaining a dry extract from *S. salicifolia* shoots that has well-pronounced anti-inflammatory, diuretic, and antioxidant activities. In that study, the dry extract of *S. salicifolia* at a dose of 100 mg/kg had an anti-inflammatory effect, as evidenced by a decrease in the degree of tissue alteration and an enhancement of recovery of physiological processes at the site of inflammation. The area of tissue damage on days 9 and 29 was smaller by 14.9% and 19.9%, respectively. The tested dose of the dry extract of *S. salicifolia* had an antiexudative effect, reducing the swelling of the animals’ paw by 36.5% as compared to a control group [149].

### 4.2. Antioxidant Activities

Polyphenols of *Spiraea* are promising natural antioxidants for the food and pharmaceutical industries. Information on the antioxidant effects of *Spiraea* is compiled in Table 3. The investigation into the antioxidant potential of most spireas has been based on the DPPH method. Trolox [150,151] is used more often as a comparison sample; ascorbic acid [148] and *n*-propyl gallate are employed less often [29]. In some research articles, the antioxidant activity of a reference compound is not specified [80]. An assessment of the cytotoxicity of extracts or their fractions from spireas has been conducted only in the studies where, in addition to the antioxidant activity, an anti-inflammatory effect was evaluated [24,148].

A crude extract of *S. canescens* and its partitioned fractions (ethyl acetate and butanolic) have been found to exert potent actions in radical-scavenging and superoxide anion-scavenging assays, while a moderate activity was found in a Fe^+2^-chelating assay [29]. The activity of the extract and fractions is comparable to the activity of the standard [*n*-propyl gallate: DPPH radical-scavenging activity (90.13%) and superoxide anion-scavenging activity (91.04%)]. In addition, pure compounds were isolated from *S. canescens*, and their antioxidant activity was evaluated. The assay results revealed a higher scavenging activity of quercetin and cyclolignans [(+)-isolariciresinol 3a-O-b-D-glucopyranoside and (+)-lyoniresinol 3a-O-b-D-glucopyranoside] toward the DPPH and superoxide anion radicals. Among them, quercetin showed a potent activity against superoxide anions (IC_50_ = 68.11 µM), which was stronger than that of the standard compound *n*-propyl gallate (IC_50_ = 106.23 µM). In addition, quercetin has been found to be a potent scavenger of DPPH radicals (IC_50_ = 50.30 µM); these findings point to its strong antioxidant properties [29].

A methanolic extract of *S. fritschiana* has an antioxidant activity, with RC_50_ of 76.61 µg/mL [148]. The antiradical activity of this extract is approximately 14-fold lower than that in the control (ascorbic acid, RC_50_ = 5.37 µg/mL). As a result of measuring the reducing ability of a spirea extract at 25–500 μg/mL, it was confirmed that the absorption value (0.13–1.8) increased significantly as the concentration of the sample increased. The extract showed approximately 50% reducing capacity as compared to butylated hydroxytoluene (absorbance = 0.77) used as a control at 100 μg/mL. Considering that only a crude extract from the spirea aerial part was tested above, it can be said that its antiradical activity is relatively high. Furthermore, a high total level of phenolic compounds (212.78 µg gallic acid/mg) and flavonols (66.84 µg quercetin/mg) was found [148].

Seeds of native and naturalized plants currently growing in the Mississippi River Basin of the United States have been evaluated as potential new sources of antioxidant activity [151]. Antioxidant levels of 158 studied plants ranged from 2.40 to 261.38 μM Trolox/100 g. The screening of extracts from the plants’ seeds for antiradical activity showed that a methanolic extract from *S. tomentosa* seeds (141.31 µmol Trolox/100 g) has a high antioxidant activity [151].

Total antioxidant levels in extracts from leaves and inflorescences of nine representatives of spireas growing in the Far East of Russia have been investigated by the operative amperometric method [152]. In that study, among the Far Eastern representatives of this genus, *S. betulifolia* (2.79 mg/g in an aqueous extract from inflorescences) and *S. beauverdiana* (2.54 mg/g in an aqueous extract from leaves and 2.11 mg/g in an aqueous-ethanolic extract from leaves) (section *Calospira*), *S. humilis* (1.61 mg/g in the aqueous extract from inflorescences) and *S. salicifolia* (0.82 mg/g in the aqueous extract from leaves) (section *Spiraria*), *S. pubescens* (0.72 mg/g in the aqueous-ethanolic extract from leaves), and *S. media* (0.59 mg/g in the aqueous extract from leaves) (section *Chamaedryon*) were found to be promising antioxidant materials. Spireas probably contain water-soluble antioxidant compounds of the phenolic type because the antioxidant activity of aqueous extracts from the leaves and inflorescences is higher (0.16–2.79 mg/g) than that of water-alcohol extracts (0.06–2.54 mg/g). The antioxidant activity in the leaves of spireas is generally higher than that in inflorescences. A significant positive correlation was observed between the antioxidant activity of aqueous extracts from the organs of *Spiraea* and the level of oxycinnamic acids [152]. In a study on antioxidant activity of aqueous and aqueous-ethanolic extracts from *S. media* leaves and inflorescences collected in a natural population (Ust-Kulomsky District, Komi Republic), it was found that aqueous extracts of *S. media* leaves have a lower radical-binding activity than do the aqueous-ethanolic extracts [150]. At the same time, the radical-binding activity of the aqueous-ethanolic extract of leaves was greater than that of inflorescences. In that study, the aqueous-ethanolic extract of leaves showed the greatest radical-binding activity among *S. media* extracts. Its IC_50_ was 4.1-fold higher than that of a standard (Trolox, IC_50_ = 1.18 mg/mL). A strong correlation between the radical-binding activity and concentration of polyphenols was found in the extracts (*r* = –0.80, *p* < 0.05). In the same work, the assay for the ability to chelate Fe^2+^ revealed that all the extracts are 77.0–133.7-fold less active than the chelator dipyridyl (EC_50_ = 0.13 mg/mL). The aqueous-ethanolic extract of inflorescences (13.75 mg/mL) had greater chelating activity than did the aqueous extract [150].

The high antioxidant potential of *S. prunifolia* var. *simpliciflora* has been confirmed many times. The extract has been found to have a high activity similar to that of superoxide dismutase and a high total level of phenolic compounds (56.7 µg gallic acid/mg) and flavonols (15.1 µg rutin/mg) [80]. In a study by Lee et al. [24], a methanolic extract of *S. prunifolia* var. *simpliciflora* leaves showed a potent DPPH radical-scavenging activity and suppressed reactive oxygen species production in tumor necrosis factor-stimulated NCI-H292 cells. In addition, *S. prunifolia* var. *simpliciflora* effectively attenuated lipid peroxidation and restored glutathione concentration in the lung tissues of a mouse model of LPS-induced acute lung injury and in tumor necrosis factor-stimulated NCI-H292 cells [24].

### 4.3. Antiviral Activity

An in vitro study of antiviral activity was performed on 14 plant species of the genus *Spiraea* growing in Asian Russia (Table 3) [153]. In that study, the inhibition of the replication of human influenza virus A/Aichi/2/68 (H3N2) and avian influenza virus A/chicken/Kurgan/05/2005 (H5N1) in MDCK cell culture was evaluated for extracts from the aerial part, at their highest tolerable concentrations. It was revealed that all aqueous-ethanolic extracts of the tested *Spiraea* species have an antiviral effect of various sizes. Virus neutralization indices (NIs) for these samples ranged from 0.5 to 4.25 lg toward both strains of the influenza virus. In this project, of all the tested samples, the aqueous-ethanolic extract of *S. hypericifolia* had the greatest antiviral effect (NI was 4.0 lg for human influenza virus and 4.25 lg for avian influenza virus), and this species is in the group of the least toxic *Spiraea* plants (highest tolerable concentrations for cultured MDCK cells: 0.5 mg/mL). The following plants were less potent but promising against avian influenza A/H5N1 virus: *S. alpina* and *S. crenata* (NI for these samples was 3.25 lg) as well as *S. dahurica*, *S. aquilegifolia*, *S. betulifolia*, *S. media*, *S. salicifolia*, *S. pubescens*, and *S. elegans* (NI = 2.25 to 2.75 lg), whereas against human influenza virus A/H3N2, the promising species were *S. pubescens*, *S. betulifolia*, *S. media*, *S. salicifolia*, and *S. dahurica* (NI = 2.0 to 3.0 lg) [153].

### 4.4. Antimicrobial and Antibacterial Activities

To date, all studies on the antimicrobial and antibacterial activities of spireas have been conducted in vitro (Table 3). A methanolic extract from *S. tomentosa* seeds has an inhibitory effect on four microorganisms: *Staphylococcus aureus*, *Pseudomonas aeruginosa*, *E. coli*, and *C. albicans* [151]. Nevertheless, in that report, the antibacterial activity of the *S. tomentosa* seed extract was lower than that of an antibiotic called ticarcillin (inhibition zone of 47 mm against *S. aureus*, 32 mm against *E. coli*, and 25 mm against *P. aeruginosa*), which served as a positive control. Furthermore, the antifungal activity of the extract against *C. albicans* was less than that of the antifungal mixture of essential oils “RC” from Young Living (inhibition zone of 15 mm), which was used as a positive control. It should be pointed out that *S. alba* manifested no antimicrobial activity [151]. Because the researchers have not detected a high correlation between antimicrobial and antioxidant activities in these plants, it is likely that the antimicrobial action is exerted by nonphenolic substances that are recognized as antioxidants.

Moderate antibacterial activities have been found in extract fractions (of different polarity) from the aerial part of *S. chamaedryfolia*. Three fractions showed an antibacterial activity against gram-positive and gram-negative bacterial strains, and one fraction exerted an antibacterial activity against methicillin-resistant *S. aureus* (MRSA). Solvent-soaked paper discs served as negative controls. In the fractions from the aerial part of *S. chamaedryfolia*, alkaloids were found that are possibly responsible for the antibacterial activity of this species [122,154]. Because these studies did not assess the antibacterial activity of alkaloids and other *Spiraea* species (*S. crenata*, *S. media*, *S. salicifolia*, *S. nipponica*, *S. × vanhouttei*, and *S. × billardii*), which do not contain alkaloids as shown in this project, it is not possible to draw a conclusion about the antibacterial activity of *S. chamaedryfolia* alkaloids.

In an ethanolic extract from the leaves of *S. thunbergii* J. Lee et al. [89] have revealed a high antimicrobial activity against *E. coli*, and the same was done for a butyrolactone [S-(-)-tulipalin B] isolated from the leaves of *S. thunbergii*. The researchers stated that the methylene group possibly is key for the antibacterial activity of this compound, and the hydroxyl group may have a synergistic effect with the methylene group [89].

### 4.5. Protistocidal Activity

The protistocidal effect of spireas has been assessed in vitro. There are data only on five species of *Spiraea* (Table 3). Extracts from the branches of *S. aquilegifolia* and *S. dahurica* have a protistocidal effect, causing almost instantaneous death of protozoa (the *S. media* extract does this within 5 min, and the *S. salicifolia* extract within 35 min). It was also revealed that the extracts from the roots of *S. aquilegifolia* and branches of *S. chamaedryfolia* do not have a protistocidal activity. Experiments intended to identify the active substances responsible for the protistocidal action are not mentioned in that article [155].

### 4.6. Antifungal Activity

Spireas also show antifungal activity. Antimycotic activity has been found in *S. prunifolia* (Table 3). Extracts from the leaves of *S. prunifolia* at various concentrations are reported to be effective at reducing the growth of the mycelium of seven fungi that cause the rot of tomatoes and eggplant fruits [156]. In that work, a high concentration (7 mg/mL) was found to be more effective than a low concentration (2 and 5 mg/mL). An ethanolic extract of *S. prunifolia* showed the highest antimycotic activity against *R. solani* and the weakest inhibitory effect against the growth of *Alternaria alternata*. An aqueous extract of *S. prunifolia* showed the strongest mycelial inhibition in *Aspergillus niger* and the weakest inhibitory effect on *A. alternata* [156]. Extracts from *S. prunifolia* can be used as a new natural fungicide for the management of fungal rot pathogens. Nonetheless, further studies on *S. prunifolia* extracts are needed regarding active ingredients and possible activities against other pathogenic fungi.

It has been demonstrated that different fractions of the ethanolic extract from *S. alpina* leaves inhibit mycelium growth of the fungus causing late rice sheath blight and the fungus causing corn leaf blight (Table 3). In one study [88], the ethyl acetate fraction was found to be most effective against *R. solani* and *E. turcicum* (dose 0.3 mg/mL, inhibition rates 47.4% and 40.8%, respectively). As the extract was purified, the scientists were able to isolate highly polar substances that possess the highest antifungal activity [88]. Further research by the scientists [85] led to the isolation of a new antifungal compound. The major fungitoxic ingredient of *S. alpina* turned out to be a new diacylated sugar, structurally identified as 6-O-(3′,4′-dihydroxy-2′-methylenbutyryl)-1-O-trans-cinnamoyl-β-D-glucopyranose. This compound at 0.1 mg/mL can inhibit the growth of *R. solani* and *E. turcicum* by 87.6% and 63.2%, respectively. In that study, pseudomycin A served as a reference compound. The antifungal activities against *R. solani* were similar between the isolated compound and the reference substance [85]. It is possible that this compound and extracts from *S. alpina* also have an insecticidal activity because the molecule of the isolated compound contains a relevant moiety, namely, a derivative of α-methylene-γ-butyrolactone [83]. Similar compounds isolated from *S. thunbergii* have shown insecticidal activity.

## 5. Other Beneficial Properties of *Spiraea*

Spireas also have other useful effects. Ethanolic extracts of *S. alpina* leaves have an anticancer effect [157] (Table 3). A methanolic extract of *S. cantoniensis* protects soft steel against corrosion [158]. The ability of spireas to tolerate increased concentrations of pollutants under urban conditions has been demonstrated using *S. × vanhouttei* as an example [159]. Spireas are resistant to high soil salinity [160]. *S. trilobata* leaves hold promise as an insect repellant of plant origin [161]. After an analysis of the chemical composition of flowers, leaves, and branches of 11 spireas growing in China, recommendations have been made regarding their use as tonic drinks of various quality categories, and the timing has been clarified for phenological periods most suitable for harvesting these plants [162,163].

## 6. Conclusions

The present review shows that spireas have been a subject of various scientific studies. Chemical analysis has resulted in the isolation and identification of both known compounds and previously unknown secondary metabolites in the aerial part, leaves, inflorescences, and stems of spireas. Pharmacological studies confirm the ethnomedical significance of spireas and have revealed new beneficial properties of these species. The identification of biological activities of individual compounds from spireas is also suggestive of strong effects of the whole plant. It would be worthwhile to search for species with pronounced antitumor, anti-inflammatory, neuroprotective, antiviral, and other activities among representatives of spireas and to seek their further adoption by conventional medicine. Additionally, phytochemical studies on spireas are beginning to address their in vitro culture, which has great prospects for scaling up the synthesis of some desired substances. Thus, the above information on the chemical composition and biological activities of spireas points to a fairly strong therapeutic potential. It is likely that studies on other species of this genus will yield new results that are practically useful.

## Figures and Tables

**Table 1:** Flavonoids of spireas.

ID	Compound		Species, Parts of Plant	References
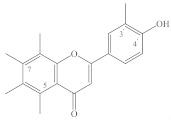 Apigenin type				
**1**	Apigenin (5,7,4′-trihydroxyflavone)		*S. hypericifolia* (S)	[39]
**2**	Apigenin-5-*O*-β-D-glucopyranoside		*S. hypericifolia* (S)	[39]
**3**	Apigenin-7-*O*-β-D-glucopyranoside		*S. canescens* (WP)	[29]
**4**	Vitexin (apigenin-8-*C*-glucoside)		*S. salicifolia* (L, Fl)	[40]
**5**	Vicenin (apigenin 6,8-di-C-glucoside)		*S. salicifolia* (L, Fl)	[40]
**6**	Sparin C (3′-methylapigenin-7-β- D-glucopyranoside)		*S. brahuica* (WP)	[30]
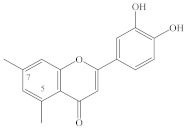 Luteolin type				
**7**	Luteolin (5,7,3′,4′-tetrahydroxyflavone)		*S. salicifolia* (L, Fl) *S. nipponica* (L, Fl, S) *S. hypericifolia* (S)	[39,40,41]
**8**	Luteolin-5-*O*-β-D-glucopyranoside		*S. hypericifolia* (S)	[39]
**9**	Luteolin-7-β-D-glucopyranoside		*S. brahuica* (WP) *S. salicifolia* (L, Fl)	[30,40]
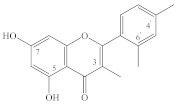 Kaempferol type				
**10**	Kaempferol (3,5,7,4′-tetrahydroxyflavone)		*S. beauverdiana* (L) *S. betulifolia* (L) *S. bumalda* (L) *S. chamaedryfolia* (L) *S. crenata* (L, Fl) *S. dahurica* (L) *S. douglasii* (L) *S. elegans* (WP) *S. flexuosa* (L) *S. humilis* (L) *S. hypericifolia* (L) *S. media* (L, Fl) *S. pubescens* (L) *S. salicifolia* (L, Fl) *S. sericea* (L) *S. schlothauerae* (L) *S. trilobata* (L) *S. ussuriensis* (L, WP)	[40,42,43,44,45,46,47,48,49]
**11**	Astragalin (kaempferol-3-*O*-glucoside)		*S. aemiliana* (L) *S. beauverdiana* (L) *S. betulifolia* (L) *S. crenata* (L, Fl) *S. formosana* (S) *S. media* (L, Fl) *S. salicifolia* (L) *S. prunifolia* var. *simpliciflora* (WP)	[31,34,42,43,45,49,50,51,52]
**12**	6″-Caffeoyl-astragalin		*S. salicifolia* (L)	[37]
**13**	Kaempferol-4′-glucoside		*S. salicifolia* (FS)	[32]
**14**	Trifolin (kaempferol-3-*O*-β-D-galactoside)		*S. salicifolia* (L) *S. prunifolia* var. *simpliciflora* (L, WP)	[31,37]
**15**	Nicotiflorin (kaempferol-3-*O*-rutinoside)		*S. salicifolia* (FS)	[32]
**16**	Robinin (kaempferol-3-*O*-robinoside- 7-*O*-rhamnoside)		*S. salicifolia* (L, Fl)	[40]
**17**	Kaempferol-3-*O*-(6″-caffeoyl)- β-D-glucopyranoside		*S. cantoniensis* (Fl) *S. salicifolia* (FS)	[32,53]
**18**	Tiliroside (kaempferol-3-*O*-(6″-*O*-*p*-coumaroyl)- -glucoside)		*S. formosana* (Fr) *S. salicifolia* (L, Fl, FS)	[32,37,54,55]
**19**	Kaempferol-3-*O*-(6″-caffeoyl)- -β-D-galactopyranoside		*S. cantoniensis* (Fl) *S. salicifolia* (L, F, FS)	[32,53]
**20**	Prunifolianoside B (kaempferol- 3-*O*-α-L-arabinopyranosyl- (1→6)-(2″-*O*-acetyl-β-D-galactopyranoside))		*S. prunifolia* var. *simpliciflora* (WP)	[31]
**21**	6′-*O*-(4″-Methoxy-*trans*-cinnamoyl)- kaempferol-3-β-D-glucopyranoside		*S. canescens* (WP)	[29]
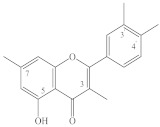 Quercetin type				
**22**		Quercetin (3,5,7,3′,4′-pentahydroxyflavone)	*S. aemiliana* (L) *S. beauverdiana* (L) *S. betulifolia* (L) *S. bumalda* (L) *S. chamaedryfolia* (L) *S. crenata* (L, Fl) *S. dahurica* (L) *S. douglasii* (L) *S. elegans* (WP) *S. formosana* (S) *S. flexuosa* (L) *S. humilis* (L) *S. hypericifolia* (L) *S. media* (L, Fl) *S. pubescens* (L) *S. salicifolia* (L, Fl) *S. sericea* (L) *S. schlothauerae* (L) *S. trilobata* (L) *S. ussuriensis* (L, WP)	[34,39,40,42,44,45,46,47,48,49,56,57,58]
**23**		Taxifolin (dihydroquercetin, 2,3-dihydroquercetin)	*S. aemiliana* (L) *S. beauverdiana* (L) *S. betulifolia* (L) *S. salicifolia* (L, Fl)	[40,49]
**24**		Rhamnetin (7-*O*-methylquercetin)	*S. salicifolia* (L)	[32]
**25**		Rhamnetin-3-*O*-β-D-glucopyranoside	*S. salicifolia* (L)	[32]
**26**		Spiraearhamnin A (rhamnetin-3-*O*-(6″-*O*-*p*-coumaroyl)- -β-D-glucopyranoside)	*S. salicifolia* (L)	[32]
**27**		Spiraearhamnin B (rhamnetin-3-*O*-(6″-*O*-*p*-caffeoyl)- β-D-glucopyranoside)	*S. salicifolia* (L)	[32]
**28**		Isorhamnetin (3′-*O*-methylquercetin)	*S. albiflora* (L) *S. betulifolia* (L) *S. crenata* (L) *S. flexuosa* (L) *S. media* (L) *S. salicifolia* (L) *S. sericea* (L) *S. ussuriensis* (L)	[48,59]
**29**		Isorhamnetin-3-*O*-α-L- rhamnopyranoside	*S. salicifolia* (L)	[37]
**30**		Isorhamnetin-3-*O*-β-D- glucopyranoside	*S. salicifolia* (L)	[37]
**31**		Narcissin (isorhamnetin-3-rutinoside)	*S. media* (L)	[59]
**32**		Prunifolianoside C (isorhamnetin-3-*O*-α-L- arabinopyranosyl- (1→ 6)-(2″-*O*-acetyl-β-D- galactopyranoside))	*S. prunifolia* var. *simpliciflora* (WP)	[31]
**33**		Rhamnazin (7,3′-di-*O*-methylquercetin)	*S. brahuica* (WP)	[30]
**34**		Spiraeoside (quercetin-4′-*O*-β-D- glucopyranoside)	*S. salicifolia* (FS)	[32,52]
**35**		Isoquercitrin (quercetin-3-*O*-glucopyranoside)	*S. aemiliana* (L) *S. aquilegifolia* (L) *S. canescens* (WP) *S. chamaedryfolia* (L) *S. formosana* (S) *S. hypericifolia* (L) *S. media* (L, Fl) *S. prunifolia* (L) *S. prunifolia* var. *simpliciflora* (WP) *S. salicifolia* (L)	[29,32,34,39,42,43,49,50,51,52,56,58,59,60,61,62]
**36**		6″-Caffeoyl-isoquercitrin- 1-*O*-*p*-hydroxybenzoyl- 6-*O*-coumaroyl-β-D-glucopyranoside	*S. salicifolia* (L)	[37]
**37**		Hyperoside (quercetin-3-β-D-galactoside)	*S. aemiliana* (L) *S. aquilegifolia* (L) *S. beauverdiana* (L) *S. betulifolia* (L) *S. chamaedryfolia* (L) *S. crenata* (L, Fl) *S. formosana* (S) *S. hypericifolia* (L) *S. media* (L, Fl) *S. salicifolia* (L, Fl, FS) *S. schlothauerae* (L) *S. trilobata* (L) *S. prunifolia* var. *simpliciflora* (WP)	[31,32,34,42,43,45,47,49,50,51,56,58,59,61,63,64]
**38**		Hyperoside-6″-caffeoyl	*S. salicifolia* (L)	[37]
**39**		Quercitrin (quercetin-3-*O*-α-L-rhamnoside)	*S. salicifolia* (FS)	[32]
**40**		Avicularin (quercetin-3-*O*-α-L-arabinoside)	*S. aemiliana* (L) *S. aquilegifolia* (L) *S. beauverdiana* (L) *S. betulifolia* *S. crenata* (L, Fl) *S. hypericifolia* (L) *S. media* (L) *S. salicifolia* (L, B)	[39,42,43,45,49,50,51,52,56,58,59,61]
**41**		Quercetin– 3-*O*-α-L- arabinofuranoside	*S. bumalda* (L)	[44]
**42**		Quercetin–3-*O*-α-L- rhamnofuranoside	*S. bumalda* (L)	[44]
**43**		Miquelianin (quercetin-3-*O*-β-D- glucuronide)	*S. salicifolia* (FS)	[32]
**44**		Quercetin-3-*O*-(6″-*O*-α-L- arabinopyranosyl)-β-D- galactopyranoside	*S. salicifolia* (L)	[42,63,64]
**45**		Rutin (quercetin-3-*O*-rutinoside)	*S. aemiliana* (L) *S. aquilegifolia* (L) *S. beauverdiana* (L) *S. betulifolia* (L) *S. hypericifolia* (L) *S. media* (L, Fl) *S. salicifolia* (L, B, FS) *S. trilobata* (L) *S. nipponica* (L, Fl, S)	[32,41,43,49,50,51,52,57,61]
**46**		Helichrysoside (quercetin-3-*O*-(6″-*O*-*p*- coumaroyl)-glucoside)	*S. salicifolia* (FS)	[32]
**47**		Quercetin-3-*O*-(6″-caffeoyl)- β-D-galactopyranoside	*S. cantoniensis* (F) *S. salicifolia* (FS)	[32,53]
**48**		Quercetin-3-*O*-(6″-caffeoyl)- β-D-glucopyranoside	*S. salicifolia* (FS)	[32]
**49**		Spireasalicin (quercetin-3-*O*-[6″-(4′′′-hydroxy- 2′′′-methylenebutyroyl)]- β-D-glucopyranoside)	*S. salicifolia* (FS)	[32]
**50**		Prunifolianoside A (quercetin 3-*O*-α-L- arabinopyranosyl-(2″-*O*-acetyl- β-D-galactopyranoside))	*S. prunifolia* var. *simpliciflora* (WP)	[31]
**51**		Quercetin 3-*O*-(2′′′-*O*-caffeoyl- α-L-arabinopyranosyl)- (1→6)-β-D-galactopyranoside	*S. prunifolia* var. *simpliciflora* (WP)	[31]
**52**		Hesperidin (hesperetin-7-rutinoside)	*S. salicifolia* (Fl)	[40]
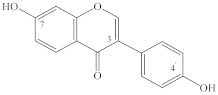 Isoflavone type				
**53**	Daidzein (7,4′-dihydroxyisoflavone)		*S. nipponica* (L, Fl, S)	[41]
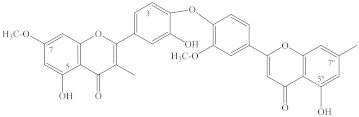 Dimeric-sparin type				
**54**	Sparin A (3,5-dihydroxy-2-{3-hydroxy- 4-[2-methoxy-4-(3,5,7-trihydroxy- 4-oxo-4H-1-benzopyran-2-yl) phenoxy]phenyl}-7-methoxy- 4H-1-benzopyran-4-one)		*S. brahuica* (WP)	[30]
**55**	Sparin B 2-{4-[4-(3,5-Dihydroxy-7-methoxy- 4-oxo-4*H*-1-benzopyran-2-yl)- 2-methoxyphenoxy]-3-hydroxyphenyl} -5 hydroxy-3,7-dimethoxy-4*H*-1- benzopyran-4-one)		*S. brahuica* (WP)	[30]
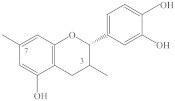 Catechin type				
**56**	(+)-Catechin		*S. hypericifolia* (S) *S. nipponica* (L, Fl, S) *S. prunifolia* (L) *S. salicifolia* (L)	[32,41,52,65,66,67,68]
**57**	(+)-Catechin-3-*O*-β-D-xylopyranose		*S. prunifolia* (L)	[60]
**58**	(+)-Catechin-7-α-L-rhamnofuranoside		*S. hypericifolia* (S)	[65,66,67,68]
**59**	(+)-Catechin-7-α-L-rhamnopyranoside		*S. hypericifolia* (S)	[65,66,67,68]
**60**	(+)-Catechin-7-α-L-arabinofuranoside		*S. hypericifolia* (S)	[65,66,67,68]
**61**	(+)-Catechin-7-α-L-xylopyranoside		*S. hypericifolia* (S)	[65,66,67,68]
**62**	(−)-Epicatechin		*S. hypericifolia* (S) *S. nipponica* (L, Fl, S) *S. prunifolia* (L) *S. salicifolia* (S)	[32,41,52,65,66,67,68]
**63**	(−)-Epigallocatechin		*S. salicifolia* (S)	[52]

B: branches; Fl: flowers; FS: flowering shoots; L: leaves; S: stem; T: twig; WP: whole plant.

**Table 2:** Phenolic acids of spireas.

ID	Compound	Species, Parts of Plant	References
**64**	Gallic	*S. chamaedryfolia* (L) *S. media* (L) *S. salicifolia* (L, Fl)	[32,40,58]
**65**	Syringic	*S. crenata* (S, R) *S. formosana* (S) *S. media* (L) *S. nipponica* (Fl, S)	[34,41,45,48]
**66**	Protocatechuic	*S. chamaedryfolia* (L) *S. hypericifolia* (L) *S. media* (L) *S. salicifolia* (FS)	[32,58,75]
**67**	Vanillic	*S. brahuica* (WP) *S. chamaedryfolia* (L) *S. crenata* (S, R) *S. formosana* (S) *S. hypericifolia* (L) *S. media* (L) *S. nipponica* (Fl, S)	[34,41,45,58,77]
**68**	Ellagic	*S. beauverdiana* (L) *S. betulifolia* (L)	[49,78]
**69**	Gentisic	*S. crenata* (S, R) *S. media* (L)	[45,48]
**70**	5-*O*-Galloylquinic	*S. salicifolia* (FS)	[32]
**71**	Cinnamic	*S. aemiliana* (L) *S. beauverdiana* (L) *S. betulifolia* (L, WP) *S. brahuica* (WP) *S. chamaedryfolia* (L) *S. crenata* (S, R) *S. media* (L) *S. nipponica* (L, Fl, S) *S. salicifolia* (L) *S. ussuriensis* (WP)	[32,41,45,46,49,63,75,79]
**72**	4-Methoxycinnamic	*S. salicifolia* (L, FS)	[32,63,64]
**73**	Chlorogenic	*S. aemiliana* (L) *S. aquilegifolia* (L) *S. beauverdiana* (L) *S. betulifolia* (L, WP) *S. chamaedryfolia* (L) *S. hypericifolia* (L) *S. media* (L) *S. salicifolia* (L, Fl) *S. schlothauerae* (L)	[32,40,47,49,52,56,58,61,75]
**74**	4-*O*-Caffeoylquinic	*S. salicifolia* (FS)	[32,52]
**75**	Caffeic	*S. brahuica* (WP) *S. chamaedryfolia* (L) *S. crenata* (S, R) *S. hypericifolia* (L) *S. media* (L) *S. nipponica* (L, Fl) *S. prunifolia* (L) *S. prunifolia* var. *simpliciflora* (WP) *S. salicifolia* (L, Fl) *S. schlothauerae* (L)	[31,32,40,41,45,47,52,56,58,60,63,64,75,77]
**76**	*p*-Coumaric	*S. aemiliana* (L) *S. beauverdiana* (L) *S. betulifolia* (L, WP) *S. chamaedryfolia* (L) *S. crenata* (S, R) *S. dahurica* (L) *S. flexuosa* (L) *S. formosana* (S) *S. humilis* (WP) *S. hypericifolia* (L) *S. media* (L) *S. nipponica* (L, Fl, S) *S. prunifolia* (L) *S. salicifolia* (FS) *S. schlothauerae* (L) *S. ussuriensis* (WP)	[32,34,41,45,46,47,49,58,61,64,75,80]
**77**	*o*-Coumaric	*S. hypericifolia* (L) *S. media* (L) *S. salicifolia* (L)	[58,63,64,75]
**78**	*p*-Hydroxybenzoic	*S. aquilegifolia* (L) *S. chamaedryfolia* (L) *S. formosana* (S) *S. hypericifolia* (L) *S. media* (L) *S. nipponica* (L, S)	[34,41,56,58,61]
**79**	Ferulic	*S. chamaedryfolia* (L) *S. crenata* (S, R) *S. hypericifolia* (L) *S. media* (L) *S. nipponica* (L, S) *S. salicifolia* (L)	[40,41,45,48,56,63,64]
**80**	Veratric	*S. formosana* (S)	[34]

Fl: flowers; FS: flowering shoots; L: leaves; R: roots; S: stem; WP: whole plant.

**Table 3:** Bioactive effects of spireas: preclinical (in vitro and in vivo) studies.

	Model/Method		Extracts and Fraction	Dose or Result	References
Anti-inflammatory activity					
*S. fritschiana*	LPS-stimulated RAW 264.7 cells		Methanolic extracts of aerial part	Nitric oxide inhibition rate: 90% at 200 µg/mL	[148]
*S. media*	Rat strain Wistar with an inflammatory process caused by subcutaneous injection of 0.5 mL of 9% acetic acid into the back with simultaneous administration of a dextran solution at a dose of 300 mg/kg of body weight		Aqueous-ethanolic extract of leaves	100 mg/kg rat weight	[23]
*S. salicifolia*	Rat strain Wistar with an inflammatory process caused by subcutaneous injection of 0.5 mL of 9% acetic acid into the back with simultaneous administration of a dextran solution at a dose of 300 mg/kg of body weight		Dry extract from shoots	100 mg/kg rat weight	[149]
*S. prunifolia* var. *simpliciflora*	Murine macrophages (RAW 264.7 cells) stimulated with interferon γ, LPS, and polymyristic acetate		Methanolic extract of roots	Dosage: extract in various concentrations up to 200 mg/mL; extract significantly inhibits the formation of nitric oxide and superoxide in cells	[22]
*S. prunifolia* var. *simpliciflora*	LPS-induced inflammatory response or H_2_O_2_-induced oxidative stress in RAW 264.7 macrophagic cells		Aqueous extract of root	Extract strongly suppresses the production of nitric oxide, decreases H_2_O_2_ cytotoxicity	[80]
*S. prunifolia* var. *simpliciflora*	Tumor necrosis factor-stimulated human airway epithelial (NCI-H292) cells and mouse model of LPS-induced acute lung injury		Methanolic extract of leaves	Dosage: 10, 25, 50, and 100 μg/mL. Extract effectively inhibits airway inflammation and reactive oxygen species-mediated oxidative stress	[24]
Antioxidant activities					
*S. canescens*		DPPH assay, superoxide anion-scavenging activity assay, iron-chelating assay	Methanolic extract (ME) and ethyl acetate fraction (EAF) and butanolic fraction (ButF) of whole plants	DPPH (% radical-scavenging assay): ME (78.52%), EAF (89.10%), ButF (63.83%); Superoxide anion-scavenging activity (% radical-scavenging assay): ME (65.10%), EAF (97.03%), ButF (78.90%); iron-chelating assay (% inhibition): ME (35.05%)	[29]
*S. fritschiana*		DPPH assay, reducing power	Methanolic extract of aerial part	DPPH: RC_50_ = 76.61 µg/mL; Reducing power: absorbance 0.13–1.80	[148]
*S. beauverdiana, S. betulifolia,* *S. dahurica,* *S. flexuosa,* *S. humilis,* *S. media,* *S. pubescens,* *S. salicifolia,* *S. ussuriensis*		Amperometric method	Aqueous and aqueous-ethanolic extracts of leaves and inflorescences	Total antioxidant level (mg gallic acid/g): aqueous extracts of leaves (0.26–2.11 mg/g); aqueous extracts of inflorescences (0.16–2.79); aqueous-ethanolic extract of leaves (0.21–2.54 mg/g); aqueous-ethanolic extract of inflorescences (0.06–0.80 mg/g)	[152]
*S. media*		DPPH assay, chelation of metal ions (CA)	Aqueous and aqueous-ethanolic extracts of leaves and inflorescences	Aqueous extract of inflorescences (DPPH: IC_50_ = 14.25 mg/mL; CA: EC_50_ = 17.38 mg/mL); Aqueous extract of leaves IC_50_ = 12.91 mg/mL; CA: EC_50_ = 10.13 mg/mL; Aqueous-ethanolic extract of inflorescences IC_50_ = 10.12 mg/mL; CA: EC_50_ = 13.75 mg/mL; Aqueous-ethanolic extract of leaves IC_50_ = 4.80 mg/mL; CA: EC_50_ = 16.71 mg/mL	[150]
*S. prunifolia* var. *simpliciflora*		DPPH assay, ABTS assay, SOD (superoxide dismutase) assay	Aqueous extract of root	DPPH: IC_50_ = 320.2 mg/mL, ABTS: IC_50_ = 124.0 mg/mL, SOD: IC_50_ = 122.6 mg/mL	[80]
*S. tomentosa*		DPPH assay	Methanolic extracts of seeds	141.31 µmol Trolox/100 g	[151]
*S. tomentosa*		DPPH assay	Methanolic extract of leaves	Dosage: 10, 25, 50, and 100 μg/mL. Extract displays potent DPPH radical-scavenging activity in tumor necrosis factor–stimulated NCI-H292 cells and effectively attenuates lipid peroxidation in lung tissues	[24]
Antiviral activity					
*S. alpina,* *S. aquilegifolia,* *S. betulifolia,* *S. beauverdiana,* *S. chamaedryfolia*, *S. crenata,* *S. dahurica,* *S. elegans,* *S. flexuosa,* *S. media,* *S. hypericifolia,* *S. pubescens,* *S. salicifolia,* *S. trilobata*		Strain of avian influenza virus A/chicken/Kurgan/05/2005 (H5N1) and strain of human influenza virus A/Aichi/2/68 (H3N2) replicated in cultured MDCK cells	Aqueous-ethanolic extracts of aerial part	NI (titer of control minus titer of experiment) for human influenza virus, lg: 0.5–4.0; NI for avian influenza virus, lg: 0.75–4.25	[153]
Antimicrobial and antibacterial activity					
*S. chamaedryfolia*		disc diffusion method	Fractions with different polarity	Inhibition zone 8–13.7 mm: gram-positive *B. subtilis* (ATCC 6633), gram-positive *S. aureus* (ATCC 29213), gram-positive *Streptococcus pneumoniae* (ATCC 49619), gram-negative *Moraxella catarrhalis* (ATCC 25238), MRSA (ATCC 43300)	[122,154]
*S. tomentosa*		disc diffusion method	Methanolic extracts of seeds	Inhibition zone 13 mm: gram-positive *S. aureus* (ATCC 12600)*,* 8 mm: gram-negative *E. coli* (ATCC 8677), 7 mm: *P. aeruginosa* (ATCC 9721), and 9 mm: the yeast *C. albicans* (ATCC 10231)	[151]
*S. thunbergii*		disc diffusion method	Crude ethanolic extract of leaves	Extract has a high antibacterial activity against *E. coli*	[89]
Protistocidal activity					
*S. aquilegifolia*, *S. dahurica*, *S. media*, *S. salicifolia*		Contact method	Aqueous extract from branches	*S. aquilegifolia* and *S. dahurica* cause instant death of protozoa (*Paramecium caudatum*); *S. salicifolia* causes death within 35 min, and *S. media* within 5 min	[155]
Antifungal activity					
*S. prunifolia*		Agar well diffusion method	Ethanolic and aqueous extract of leaves at different concentrations	Inhibition zones for *Penicillium expansum*, *A. niger*, *A. alternata*, *Mucor plumbeus*, *Penicillium chrysogenum*, *Trichothecium roseum*, and *R. solani*: ethanolic extract (12.3–23.3 mm) and aqueous extract (10.0–20.0 mm)	[156]
*S. alpina*		Plate growth rate method	Different fractions of ethanolic extract from leaves	Inhibitory rate of fractions: 13.9–47.4% for *R. solani* and 12.1–40.8% for *E. turcicum*	[85,88]
Anticancer effect					
*S. alpina*	Human liver cancer BEL-7402 cells, colon cancer HCT-8 cells, and lung carcinoma A-549 cells		Ethanolic extracts (35–70%) of leaves	Extract of *S. alpina* has some inhibitory action on human liver cancer cell line BEL-7402, colon cancer HCT-8 cells, and lung carcinoma A-549 cells	[157]

## Data Availability

Not applicable.

## References

[1] Potter D., Eriksson T., Evans R.C., Oh S., Smedmark J.E.E., Morgan D.R., Kerr M., Robertson K.R., Arsenault M., Dickinson T.A. (2007). Phylogeny and classification of Rosaceae. Plant Systemat. Evol..

[2] Hummer K.E., Janick J., Folta K.M., Gardiner S.E. (2009). Rosaceae: Taxonomy, economic importance, genomics. Genetics and Genomics of Rosaceae.

[3] Garcia-Oliveira P., Fraga-Corral M., Pereira A.G., Lourenço-Lopes C., Jimenez-Lopez C., Prieto M.A., Simal-Gandara J. (2020). Scientific basis for the industrialization of traditionally used plants of the Rosaceae family. Food Chem..

[4] Shulgina V.V., Sokolov S.Y. (1954). Genus *Spiraea* L.. Trees and Shrubs of the USSR—Native, Cultivated or Promising for Introduction.

[5] Lu L.T., Crinan A., Wu Z.Y., Raven P.H. (2003). Spiraea Linnaeus. Flora of China.

[6] The Plant List (2013) Version 1.1. https://www.theplantlist.org.

[7] Yu S.X., Gadagkar S.R., Potter D., Xu D.X., Zhang M., Li Z.Y. (2018). Phylogeny of *Spiraea* (Rosaceae) based on plastid and nuclear molecular data: Implications for morphological character evolution and systematics. Perspect. Plant Ecol. Evol. Syst..

[8] Yü T.T., Kuan K.C. (1963). Taxa nova Rosacearum sinicarum, I. Spiraea L., Systema Spiraeae Sinicae. Acta Phytotax. Sin..

[9] Businský R. (2015). Transitive inflorescence types in Spiraea (Rosaceae-Spiraeoideae) undermine the fundamental classification concept of the genus. Phyton.

[10] Drábková L.Z., Pospíšková M., Businský R. (2017). Phylogeny and infrageneric delimitation in Spiraea (Rosaceae) inferred from AFLP markers and a comparison with morphology. Bot. J. Linn. Soc..

[11] Svyazeva O.A. (1967). Distribution of Woody Rosaceae in USSR (Especially on the Example of the Genus Spiraea). Ph.D. Thesis.

[12] Kartesz J.T. (1994). A Synonymized Checklist of the Vascular Flora of the United States, Canada, and Greenland, Second Edition, Thesaurus.

[13] Lis R.A., Flora of North America Editorial Committee (2014). Spiraea. Flora of North America.

[14] Zhang X.S., Wang B.D. (1986). Chinese Medicine Dictionary.

[15] Wu Z.Y. (1984). Index Florae Yunnannensis.

[16] Xie Z.W. (1996). Quanguo Zhongcaoyao Huibian (A Collection of Chinese Herbal Drugs).

[17] Khan S.W., Khatoon S. (2007). Ethnobotanical studies on useful trees and shrubs of Haramosh and Bugrote valleys, in Gilgit northern area of Pakistan. Pak. J. Bot..

[18] Herrick J.W. (1977). Compound Decoction of Mashed and Powdered Dried Roots Taken for Side Pain. Ph.D. Thesis.

[19] Turner N., Bouchard R., Kennedy D. (1980). Ethnobotany of the Okanagan-Colville Indians of British Columbia and Washington.

[20] Lavrenov V.K., Lavrenova G.V. (1999). Complete Encyclopedia of Medicinal Plants.

[21] Batorova S.M. (2013). Guide to Traditional Tibetan Medicinal Plants.

[22] So H.S., Park R., Oh H.M., Pae H.O., Lee J.H., Chai K.Y., Chung S.Y., Chung H.T. (1999). The methanol extract of Spiraea prunifolia var. simpliciflora root inhibits the generation of nitric oxide and superoxide in RAW 264.7 cells. J. Ethnopharmacol..

[23] Mirovich V.M., Tsyrenzhapov A.V., Krivosheev I.M. (2018). Investigation of anti-inflammatory of Spiraea media Franz Schmidt. Innov. Technol. Pharm..

[24] Lee B.W., Ha J.H., Shin H.G., Jeong S.H., Jeon D.B., Kim J.H., Park J.Y., Kwon H.J., Jung K., Lee W.S. (2020). Spiraea prunifolia var. simpliciflora attenuates oxidative stress and inflammatory responses in a murine model of lipopolysaccharide-induced acute lung injury and TNF-α-stimulated NCI-H292 cells. Antioxidants.

[25] Hao X., Shen Y., Li L., He H. (2003). The chemistry and biochemistry of Spiraea japonica complex. Curr. Med. Chem..

[26] Teng Y., Yang Q., Yu Z., Zhou G., Sun Q., Jin H., Hou T. (2010). In vitro antimicrobial activity of the leaf essential oil of Spiraea alpina Pall. World. J. Microbiol. Biotechnol..

[27] Zhang W.H., Qian H., Song Y.J., Shen T. (2017). Chemical composition, DPPH Free radical scavenging and antimicrobial activity of the essential oil and six compound isolated from *Spiraea mongolica*. Maxim. Mod. Food. Sci. Technol..

[28] Kirillov V., Stikhareva T., Atazhanova G., Ercisli S., Makubayeva A., Krekova Y., Rakhimzhanov A., Adekenov S. (2021). Volatiles composition from aerial parts of the incect-pollinated and the promising medicinal plant Spiraea hypericifolia L. growing wild in Northern Kazakhstan. Nat. Prod. Sci..

[29] Choudhary M.I., Naheed N., Abbaskhan A., Ali S. (2009). Hemiterpene glucosides and other constituents from Spiraea canescens. Phytochemistry.

[30] Mughal U.R., Mehmood R., Malik A., Ali B., Tareen R.B. (2012). Flavonoid constituents from Spiraea brahuica. Helv. Chim. Acta.

[31] Yean M.H., Kim J.S., Kang S.S., Kim Y.S. (2014). A new megastigmane glucoside and three new flavonoid glycosides from Spiraea prunifolia var. simpliciflora. Helv. Chim. Acta.

[32] Olennikov D.N., Kashchenko N.I. (2017). Spireasalicin, a new acylated quercetin glycoside from Spiraea salicifolia. Chem. Nat. Compd..

[33] Olennikov D.N., Chirikova N.K. (2018). Rhamnetin glycosides from the genus Spiraea. Chem. Nat. Compd..

[34] Wu T.S., Hwang C.C., Kuo P.C., Kuo T.H., Damu A.G., Su C.R. (2004). New neolignans from *Spiraea formosana*. Chem. Pharm. Bull..

[35] Yoshida K., Hishida A., Iida O., Hosokawa K., Kawabata J. (2010). Highly oxygenated monoterpene acylglucosides from Spiraea cantoniensis. J. Nat. Prod..

[36] Yan S.D., Yao H.L., Zhang Y.H., Gao H., Liu K., Liu Y., Dong F.y., Wang W. (2016). Chemical constituents from *Spiraea salicifolia*. Chin. Trad. Herb. Drugs..

[37] Kashchenko N.I., Chirikova N.K., Olennikov D.N. (2018). Acylated flavonoids from Spiraea genus as inhibitors of α-amylase. Rus. J. Bioorg. Chem..

[38] Kashchenko N.I., Olennikov D.N., Chirikova N.K. (2014). Ellagitannins in Rosaceous plants from the flora of Sakha (Yakutia) republic. Butl. Commun..

[39] Chumbalov T.K., Pashinina L.T., Storozhenko N.D. (1975). Flavones and their 5-glycosides from *Spiraea hypericifolia*. Chem. Nat. Compd..

[40] Krivosheev I.М., Mirovich V.M. (2012). The study of chemical composition of *Spiraea salicifolia* L. overground organs by Highly Effective Liquid Chromatography method. Sib. Med. J..

[41] Keskin S., Sirin Y., Cakir H.E., Kaya G., Keskin M. (2019). Phenolic composition and antioxidant properties of *Spiraea nipponica*. Int. J. Sci. Technol. Res..

[42] Hörhammer L., Hänsel R., Endres W. (1956). Über die flavonglykoside der gattungen Filipendula und Spiraea. Arch. Pharm..

[43] Blinova K.F., Stuckey K.L. (1964). Pharmacognostic research of medicinal plants of Tibetan medicine. Vopr. Farmakogn..

[44] Sennikov G.A., Makarova G.V. (1969). Polyphenol compounds of Spiraea Bumalda. Farmatsevtychnyi Zhurnal.

[45] Paris R.R., Murgu L. (1970). Polyhenolic compounds of *Spiraea crenata*. Plant Med. Phytother..

[46] Kostikova V.A. (2013). Research of phenolic compounds in plants of genus Spiraea L. of Russian Far East by methods of HPLC. Tambov Univ. Rev..

[47] Kostikova V.A. (2018). Phenolic compounds in *Pentactina schlothauerae* (=*Spiraea schlothauerae*). Proc. Univ. Appl. Chem. Biotechnol..

[48] Karpova E.A., Lapteva N.P. (2014). Phenolic compounds in taxonomy of the genus Spiraea L.. Turczaninowia.

[49] Kostikova V.A., Kuznetsov A.A., Tishchenko E.D., Fayzylkhakova A.N. (2019). Chemotaxonomic study of *Spiraea aemiliana* compared to the closely species S. betulifolia and S. beauverdiana. Acta Biol. Sib..

[50] Bandyukova V.A. (1969). Distribution of flavonoids in some families of higher plants. Rastit. Resur..

[51] Bodalski T., Cisowski W. (1969). Flavonoids in the inflorescence of Spiraea media Schm. Diss. Pharm. Pharmacol..

[52] Krivosheev I.M. (2014). Pharmacognostic Study of Spiraea salicifolia L. Growing in Eastern Siberia. Ph.D. Thesis.

[53] Yoshida K., Hishida A., Iida O., Hosokawa K., Kawabata J. (2008). Flavonol caffeoylglycosides as alpha-glucosidase inhibitors from Spiraea cantoniensis flower. J. Agric. Food. Chem..

[54] Lin L.C., Chou C.J., Yang L.M. (1999). Chemical constituents from the fruit of Spiraea formosana. Zhonghuá Yáoxué Zázhì (Chin. Pharm. J.).

[55] Grochowski D.M., Locatelli M., Granica S., Cacciagrano F., Tomczyk M. (2018). A review on the dietary flavonoid tiliroside. Compr. Rev. Food. Sci. Food. Saf..

[56] Storozhenko N.D. (1977). Polyphenol Compounds of *Spiraea hypericifolia* L.. Ph.D. Thesis.

[57] Zhanymkhanova P.Z., Toigambekova N.N., Duisenbaev N.K., Adekenova A.S., Mukusheva G.K., Adekenov S.M. (2012). Search of biologically active phenolic compounds in plant. Collect. Sci. Pap. Dev. Res. Mark. New Pharm. Prod..

[58] Karpova E.A., Khramova E.P. (2019). Dynamics of phenolic composition and content of representatives of the genus Spiraea L. under the conditions of transport and industrial pollution in Novosibirsk. Chem. Sustain. Dev..

[59] Karpova Е.А., Polyakova Т.А., Bochkin V.D. (2016). Flavonoids in the leaves of Spiraea media var. media and Spiraea media var. sericea (Rosaceae). Plant Life Asian Russ..

[60] Park S.H., Park K.H., Oh M.H., Kim H.H., Choe K.I., Kim S.R., Park K.J., Lee M.W. (2013). Anti-oxidative and anti-inflammatory activities of caffeoyl hemiterpene glycosides from Spiraea prunifolia. Phytochemistry.

[61] Karpova Е.А., Imetkhenova О.V. (2015). Phenolic compounds of representatives of sect. Glomerati of genus Spiraea L. of the flora of Siberia. Turczaninowia.

[62] Kim C.S., Oh J., Suh W.S., Jang S.W., Subedi L., Kim S.Y., Choi S.U., Lee K.R. (2017). Investigation of chemical constituents from *Spiraea prunifolia* var. *simpliciflora* and their biological activities. Phytochem. Lett..

[63] Bate-Smith E.C. (1962). The phenolic constituents of plants and their taxonomic significance. Bot. J. Linn. Soc..

[64] Ahn B.T., Oh K.J., Park S.K., Chung S.G., Cho E.H., Kim J.G., Ro J.S., Lee K.S. (1996). Phenolic compounds from leaves of *Spiraea salicifolia*. Kor. J. Pharmacogn..

[65] Chumbalov T.K., Pashinina L.T., Storozhenko N.D. (1974). Flavans of *Spiraea hypericifolia*. Chem. Nat. Compd..

[66] Chumbalov T.K., Pashinina L.T., Storozhenko N.D. (1974). Chromatographic study of *Spiraea hypericifolia*. Chem. Chem. Technol..

[67] Chumbalov T.K., Pashinina L.T., Storozhenko N.D. (1976). Catechin 7-rhamnoside from *Spiraea hypericifolia*. Chem. Nat. Compd..

[68] Chumbalov T.K., Pashinina L.T., Storozhenko N.D. (1976). Catechin-7-xyloside from *Spirea hypericifolia*. Chem. Nat. Compd..

[69] Ozipek M., Caliş I., Ertan M., Rüedi P. (1994). Ramninoside 3-*p*-coumaroylrhamnetin from *Rhamnus petiolaris*. Phytochemistry.

[70] Manga S.S.E., Tih A.E., Abderamane B., Ghogomu R.T., Blond A., Bodo B. (2012). Flavonoid glycosides and their *p*-coumaroyl esters from *Campylospermum calanthum* leaves. Z. Naturforsch..

[71] Serebryakova V.A., Vysochina G.I. (2011). Research on the composition and content of biologically active substances of Far East representatives of genus *Spiraea* (Rosaceae). Plant Life Asian Russ..

[72] Pashinina L.T., Storozhenko N.D., Chumbalov T.K. (1976). Proanthocyanidin dimers from *Spiraea hypericifolia*. Chem. Nat. Compds..

[73] Belemets N.М., Grakhov V.P., Fedoronchuk М.М., Palamarchuk B.Z. (2014). Study on the secondary metabolites of xerophytic species of *Spiraea* L. (Rosaceae) genus from Ukraine flora. J. VN Karazin. Kharkiv Nation. Univ. Ser. Biol..

[74] Kim T.W., Lee Y.M. (1993). Taxonomic Studies on the Genus *Spiraea* in Korea Based on Flavonoid Characteristics. Bull. Seoul Nat. Univ. Arboretum..

[75] Karpova E.A., Polyakova T.A. (2014). Seasonal dynamics of the composition of phenolic compounds of the leaves of *Spiraea media* var. *sericea* (Turcz.) Regel. Chem. Plant Raw Mater..

[76] Kostikova V.A., Kuznetsov A.A. (2021). Changes in the sets and levels of flavonoids and Phenolcarboxylic Acids in the Leaves of *Spiraea betulifolia* subsp. *aemiliana* (Rosaceae) during Introduction into Novosibirsk Conditions. Chem. Sustain. Dev..

[77] Shabbir S., Khan S., Kazmi M.H., Fatima I., Malik I., Inamullah F., Tareen R.B. (2020). Brahucins A and B, new triterpene lactones from *Spiraea brahuica*. J. Asian Nat. Prod. Res..

[78] Kostikova V.A. (2017). Determination of optimum conditions of extraction for investigation of composition of phenolic compounds *Spiraea betulifolia* Pall. by HPLC method. Chem. Plant Raw Mater..

[79] Shabbir S., Fatima I., Inamullah F., Mughal U.R., Khan S., Kazmi M.H., Malik A., Tareen R.B., Abbas T. (2016). Brahin, a new lipogenase inhibiting triterpene from *Spiraea brahuica*. Chem. Nat. Compd..

[80] Sim M.O., Lee H.J., Jang J.H., Lee H.E., Jung H.K., Kim T.M., No J.h., Jung J., Jung D.E., Cho H.W. (2017). Anti-inflammatory and antioxidant effects of *Spiraea prunifolia* Sieb. et Zucc. var. *simpliciflora* Nakai in RAW 264.7 cells. Korean J. Plant Res..

[81] Youn H.S., Chung B.S. (1987). Studies on the Constituents of the Roots of *Spiraea prunifolia* var. *simpliciflora*. Korean J. Pharmacog..

[82] Jang S.W., Suh W.S., Kim C.S., Kim K.H., Lee K.R. (2015). A new phenolic glycoside from *Spiraea prunifolia* var. *simpliciflora* twigs. Arch. Pharm. Res..

[83] Kim C.S., Datta P.K., Hara T., Itoh E., Horiike M. (1999). Precursor of α-methylene-γ-butyrolactone involved in the insecticidal activity of Thunberg spiraea, *Spiraea thunbergii*. Biosci. Biotechn. Biochem..

[84] Kim C.S., Hara T., Datta P.K., ItoH E., Horiike M. (1998). Insecticidal component in Thunberg spiraea, *Spiraea thunbergii*, against *Thrips palmi*. Biosci. Biotechnol. Biochem..

[85] Hou T., Teng Y., Sun Q., Yu Z. (2009). A new fungitoxic metabolite from *Spiraea alpina*. Pall. Fitoter..

[86] Hiradate S., Morita S., Sugie H., Fujii Y., Harada J. (2004). Phytotoxic *cis*-cinnamoyl glucosides from *Spiraea thunbergii*. Phytochemistry.

[87] Morita S., Hiradate S., Fujii Y., Harada J. (2005). *cis*-Cinnamoyl glucoside as a major plant growth inhibitor continued in *Spiraea prunifolia*. Plant Grow. Regulat..

[88] Teng Y., Yu Z., Cui W., Zhang X., Quan X., Sun Q., Hou T. (2009). An antifungal active component from *Spiraea alpina* to plant fungi. Sci. Agricult. Sinica.

[89] Lee J., Lee J., Lim J., Sim S., Park D. (2008). Antibacterial effects of S-(-)-tulipalin B isolated from *Spiraea thunbergii* Sieb. on Escherichia coli, major food borne pathogenic microorganism. J. Med. Plant. Res..

[90] Kim S.Y., Song N.Y., Cho J.G., Kwon J.H., Song M.C., Ahn E.M., Kang H.C., Baek N.I. (2015). Phenylglycosides from the stems of *Spiraea prunifolia* var. *simpliciflora*. Chem. Nat. Compd..

[91] Oh S.M., Choi D.J., Kim H.G., Lee J.W., Lee Y.S., Lee J.H., Lee S.E., Kim G.S., Baek N.I., Lee D.Y. (2018). Neuroprotective effects of phenolic compounds isolated from *Spiraea prunifolia* var. *simpliciflora*. J. Appl. Biol. Chem..

[92] Lee D.W., Kim J.R., Lee W.S., Cho K.H., Bae K.H., Jeoung T.S. (2003). LDL-Antioxidant activity of 6-hydroxyeugenol from *Spiraea blumei*. Proc. Conv. Pharm. Soc. Korea.

[93] Zuo G.Y., He H.P., Hong X., Shen Y.M., Hao X.J. (2005). Chemical constituents of *Spiraea japonica* var. *ovalifolia*. Acta Bot. Yunn..

[94] Yao H.L., Gao H., Yan S.D., Liu Y., Liu X.H., Zhang Y.H., Dong F.Y., Wang W. (2016). Chemical constituents from *Spiraea pubescens*. Chin. Tradit. Herb. Drugs.

[95] Sun S., Liu Y., Liu X., Zhang S., Wang W., Wang R., Hou Y., Wang W. (2019). Neolignan glycosides from *Spiraea salicifolia* and their inhibitory activity on pro-inflammatory cytokine interleukin-6 production in lipopolysaccharide-stimulated RAW 264.7 cell. Nat. Prod. Res..

[96] Nakanishi T., Iida N., Inatomi Y., Murata H., Inada A., Murata J., Frank A., Iinuma M., Tanaka T. (2004). Neolignan and flavonoid glycosides in *Juniperus communis* var. *depressa*. Phytochemistry.

[97] Oh H., Oh G.S., Seo W.G., Pae H.O., Chai K.Y., Kwon T.O., Lee Y.H., Chung H.T., Lee H.S. (2001). Prunioside A: A new terpene glycoside from *Spiraea prunifolia*. J. Nat. Prod..

[98] Oh H., Shin H., Oh G.S., Pae H.O., Chai K.Y., Chung H.T., Lee H.S. (2003). The absolute configuration of prunioside A from *Spiraea prunifolia* and biological activities of related compounds. Phytochemistry.

[99] Lee W.Y., Kim B.H., Lee Y.H., Choi H.G., Jeon B.H., Jang S.I., Kim Y.J., Chung H.T., Kim Y.S., Chai K.Y. (2004). The inhibitory effect of prunioside A acyl derivatives on NO production in RAW 264.7 cell. Bull. Korean. Chem. Soc..

[100] Jun C.S., Yoo M.J., Lee W.Y., Kwak K.C., Bae M.S., Hwang W.T., Son D.H., Chai K.Y. (2007). Ester derivatives from tannase-treated prunioside A and their anti-inflammatory activities. Bull. Korean Chem. Soc..

[101] Rathore J.S., Rathore V., Shekhawat N.S., Singh R.P., Liler G., Phulwaria M., Dagla H.R., Srivastava P., Narula A., Srivastava S. (2004). Micropropagation of woody plants. Plant Biotechnology and Molecular Markers.

[102] Khojasteh A., Metón I., Camino S., Cusido R.M., Eibl R., Palazon J. (2019). *In vitro* study of the anticancer effects of biotechnological extracts of the endangered plant species *Satureja khuzistanica*. Int. J. Mol. Sci..

[103] Kikowska M., Thiem B., Szopa A., Ekiert H. (2020). Accumulation of valuable secondary metabolites: Phenolic acids and flavonoids in different *in vitro* systems of shoot cultures of the endangered plant species—*Eryngium alpinum* L.. Plant. Cell. Tiss. Organ. Cult..

[104] Lane W.D. (1979). *In vitro* propagation of *Spirea bumalda* and *Prunus cistena* from shoot apices. Can. J. Plant Sci..

[105] Yang G., Read P.E. (1993). *In vitro* culture of Vanhoutte’s spirea explants from ‘secondary cultures’ and dormant stems forced in solutions containing plant growth regulators. Plant Cell. Tissue Organ Cult..

[106] Herrington E., McPherson J.C. (1993). Light quality growth promotion of *Spiraea nipponica*: The influence of a low photon fluence rate and transfer time to a higher fluence rate. Plant Cell. Tissue Organ Cult..

[107] Nebykov M.V., Koldar L.A., Bonyk Z.G., Trofimenko N., Belemets N. (2016). Microclonal breeding in whitish-grey meadowsweet (*Spiraea cana* Waldst. et Kit.). J. Nativ. Alien Plant Stud..

[108] Bancheva S., Delcheva M., Kikindonov T. (2019). In-situ and ex-situ conservation of *Spiraea crenata* (*Rosaceae*) in Bulgaria. Flora Mediterr..

[109] Coffin R., Taper C.D., Chong C. (1976). Sorbitol and sucrose as carbon source for callus culture of some species of the Rosaceae. Can. J. Bot..

[110] Muraseva D.S., Kostikova V.A. (2021). *In vitro* propagation of *Spiraea betulifolia* subsp. *aemiliana* (Rosaceae) and comparative analysis of phenolic compounds of microclones and intact plants. Plant. Cell. Tissue Organ Cult..

[111] Wehmer C., Hadders M., Bergmann M. (1933). Systematische Verbreitung und Vorkommen der Amide. Spezielle Analyse.

[112] Ma Y., Mao X.Y., Huang L.J., Fan Y.M., Gu W., Yan C., Huang T., Zhang J.X., Yuan C.M., Hao X.J. (2016). Diterpene alkaloids and diterpenes from *Spiraea japonica* and their anti-tobacco mosaic virus activity. Fitoterapia.

[113] Liu H.Y., Ni W., Chen C.X., Di Y.T., Hao X.J. (2009). Two new diterpenoid lactams from *Spiraea japonica* var. *ovalifolia*. Helv. Chim. Acta.

[114] Liu H.Y., Gao S., Di Y.T., Chen C.X., Lü Y., Zhang L., Zheng Q.T., Hao X.J. (2007). A novel atisane diterpenoid from *Spiraea japonica* var. *acuta*. Helv. Chim. Acta.

[115] Qin X.D., Yang S., Zhao Y., Wang L.X., Ren F.C., Wang F. (2016). Three new atisane diterpenoids from *Spiraea japonica*. Helv. Chim. Acta.

[116] Zuo G., He H., Shen Y., Xu X., Wang Z., Yan C., Hao X. (2009). Spiraeosides A and B, two new diterpenoid glucosides from *Spiraea japonica* var. *ovalifolia*. Planta Med..

[117] Fan L., Zhang Z., Shen Y., Hao X. (2004). Five diterpene alkaloids from *Spiraea japonica* (Rosaceae). Biochem. Syst. Ecol..

[118] Fan L.M., He H.P., Shen Y.M., Hao X.J. (2005). Two new diterpenoid alkaloids from *Spiraea japonica* L. f. var. *fortunei* (Planchon) Rehd. J. Integr. Plant Biol..

[119] Jin K.D. (1967). Studies on the constituents of *Spiraea koreana* Nakai. J. Korean Chem. Soc..

[120] Wu T.S., Hwang C.C., Kuo P.C., Damu A.G., Chou C.J., Chen C.F. (2002). New diterpenoid alkaloid from *Spiraea formosana*. Heterocycles.

[121] Li M., Du X.B., Shen Y.M., Wang B.G., Hao X.J. (1999). New diterpenoid alkaloids from *Spiraea frutschiana* var. *parvifolia*. Chin. Chem. Lett..

[122] Kiss T., Cank K.B., Orbán-Gyapai O., Liktor-Busa E., Zomborszki Z.P., Rutkovska S., Pučka I., Németh A., Csupor D. (2017). Phytochemical and pharmacological investigation of *Spiraea chamaedryfolia*: A contribution to the chemotaxonomy of *Spiraea* genus. BMC Res. Notes.

[123] Cheng H., Zeng F.H., Yang X., Meng Y.J., Xu L., Wang F.P. (2016). Collective total syntheses of atisane-type diterpenes and atisine-type diterpenoid alkaloids: (±)-spiramilactone B, (±)-spiraminol, (±)-dihydroajaconine and (±)-spiramines C and D. Angew. Chem..

[124] Shen Z., Chen Z., Li L., Lei W., Hao X. (2000). Antiplatelet and antithrombotic effects of the diterpene spiramine Q from *Spiraea japonica* var. *incisa*. Planta Med..

[125] Li L., Nie J., Shen Z., Wu W., Chen Z., Hao X. (2001). Neuroprotective effects in gerbils of spiramine T from *Spiraea japonica* var. *acuta*. Planta Med..

[126] Li L., Shen Y.M., Yang X.S., Zuo G.Y., Shen Z.Q., Chen Z.H., Hao X.J. (2002). Antiplatelet aggregation activity of diterpene alkaloids from *Spiraea japonica*. Eur. J. Pharm..

[127] Sheng Z.Q., Chen P., Zhang L.Y., Li D. (2004). Spiramine N-6, A novel agent of antiplatelet and anti-platelet-neutrophil interactions. Nat. Prod. Res. Dev..

[128] Mao X., Wang C., Liu W., Yu J., Sun Z., Wang M., Luo H. (2020). Anti-TMV activity of atisine-type diterpene alkaloids in *Spiraea japonica* associates with down-regulating the expression of TMV coat protein. Trends Biochem. Eng..

[129] Yan C., Huang L., Liu H.C., Chen D.Z., Liu H.Y., Li X.H., Zhang Y., Geng M.Y., Chen Q., Hao X.J. (2014). Spiramine derivatives induce apoptosis of Bax^-/-^Bax^-/-^cell and cancer cells. Bioorganic. Med. Chem. Lett..

[130] Tang D.H., Ma D., Cheng H., Li Y.L., Xu L. (2016). A bio-inspired synthetic route to the core ring systems of *Spiraea* atisine-type diterpenoid alkaloids and related diterpenes. Org. Biomol. Chem..

[131] Tanabe Y., Sinoda R., Horikoshi Y., Takahashi K. (1976). Studies on constituents of medicinal plants. XVI. The constituents of *Spiraea species*. J. Pharm. Soc. Jpn..

[132] Chou C.J., Wang C.B., Lin L.C. (1977). Triterpenoids and some other constituents from *Spiraeae formosana*. J. Chin. Chem. Soc..

[133] Woo M.H., Lee E.H., Chung S.O., Woo M.H. (1996). Constituents of *Spiraea prunifolia* var. *simpliciflora*. Korean J. Pharmacogn..

[134] Cui Z.H., Liu W., Zhang Y.H., Wu C.H., Dong F.Y., Wang W. (2011). Pentacyclic triterpenoids from *Spiraea pubescens*. Chin. J. Exp. Trad. Med. Form..

[135] Mughal U.R., Mehmood R., Malik A., Ali B., Safder M., Tareen R.B. (2012). Spiraeamide, new sphingolipid from *Spiraea brahuica*. J. Asian Nat. Prod. Res..

[136] Takeda Y., Fukumoto K., Tachibana T., Shingu T., Fujita T., Ichihara T. (1990). Monoterpene glucosides having a cross conjugated dienone system from *Spiraea cantoniensis*. Phytochemistry.

[137] Pande B.S., Krishnappa S., Bisarya S.C., Dev S. (1971). Studies in sesquiterpenes—XLVII: Cis- and trans-atlantones from *Cedrus deodara* loud. Tetrahedron.

[138] Rao A.S. (2017). Isolation, absolute configuration and bioactivities of megastigmanes or C13 isonorterpinoides. Chem. Int..

[139] Shirshova T.I., Smirnova A.N., Beshlei I.V., Ufimtsev K.G. (2019). Valuable bioactive substances of *Spiraea* (Rosaceae) species from the republic of Komi. Rastit. Resur..

[140] Ro J.S. (1982). Studies on the Constituents of the *Spirea* Plants (I)-Sterols from the Root of *Spiraea prunifolia* var. *simpliciflora*. Korean J. Pharmacog..

[141] Mirovich V.M., Krivosheev I.M. (2019). The study of chemical compositions of flowers of *Spiraea salicifolia* L. by the chromatography-mass-spectrometry method. IV Hammerman Readings: Collection of Scientific Works.

[142] Kudaibergen A.A., Dyusebaeva M.A., Ydyrys A., Feng Y., Jenis J. (2019). Investigation of chemical constituents of medical plant *Spiraea hypericifolia* L.. Int. J. Biol. Chem..

[143] Shirshova T.I., Smirnova A.N., Beshlei I.V. (2020). Biologically active compounds in leaves and inflorescences of wild and cultivated *Spiraea media* (Rosaceae) from the Komi republic. Rastit. Resur..

[144] Sorokopudov V.N., Nelasova N.V. (2015). Ascorbic acid in leaves of species of genus *Spiraea,* L. in conditions of the Belgorod area. Pomic. Small Fruits Cult. Russ..

[145] Mirovich V.M., Fedoseeva G.M., Krivosheev I.M., Konenkina T.A., Manyak V.A. (2012). The research of element structure of *Spiraea salicifolia* L. overground organs. Bull. Buryat State Univ..

[146] Huang J.H., Chen L.Z. (1991). A study of chemical contents in a mixed shrubland near Baihuashan mountain in Beijing. Chin. J. Plan. Ecol..

[147] Bakkali F., Averbeck S., Averbeck D., Idaomar M. (2008). Biological effects of essential oils—A review. Food. Chem. Toxicol..

[148] Choi E.Y., Heo S.I., Kwon Y.S., Kim M.J. (2016). Anti-oxidant activity and anti-inflammatory effects of *Spiraea fritschiana* Schneid. extract. Korean J. Med. Crop. Sci..

[149] Mirovich V.M., Krivosheev I.M., Gordeeva V.V., Tsyrenzhapov A.V. (2013). Method for Producing Agent Possessing Anti-Inflammatory, Diuretic and Antioxidant Activity. Russian Patent.

[150] Shirshova T.I., Bezmaternykh K.V., Beshlei I.V., Smirnova A.N., Oktyabr’skii O.N. (2020). Antioxidant properties of extracts of leaves and inflorescences of *Spiraea media* Franz Schmidt from the flora of Komi republic. Pharm. Chem. J..

[151] Borchardt J.R., Wyse D.L., Sheaffer C.C., Kauppi K.L., Fulcher R.G., Ehlke N.J., Biesboer D.D., Bey R.F. (2008). Antioxidant and antimicrobial activity of seed from plants of the Mississippi river basin. J. Med. Plant. Res..

[152] Kostikova V.A., Shaldaeva T.M. (2017). The antioxidant activity of the Russian Far East representatives of the *Spiraea* L. genus (Rosaceae Juss.). Russ. J. Bioorg. Chem..

[153] Kostikova V.A., Filippova E.I., Vysochina G.I., Mazurkova N.A. Antiviral activity of plants of the genus *Spiraea* (Rosaceae) growing in the Asian part of Russia. Proceedings of the International Conference Dedicated to the 70th Anniversary of Central Siberian Botanical Garden “Preserving the Diversity of Flora in the Botanical Gardens: Tradition, Modernity and Perspectives”.

[154] Kiss T. (2017). Phytochemical, Pharmacological and Toxicological Studies of Alkaloid-and Sesquiterpene Lactone-Containing Medicinal Plants. Ph.D. Thesis.

[155] Gammerman А.P., Blinova K.F., Badmaev A.N., Drobotko V.G. (1967). Antimicrobial activity of medicinal plants of Tibet. Phytoncides.

[156] Koka J.A., Bhat M.Y., Wani A.H. (2020). Allelopathic effect of leaf extracts of *Punica granatum* and *Spiraea prunifolia* against post-harvest rot of tomato and brinjal. J. Drug Deliv. Therapeut..

[157] Kong Q. (2014). Application of Spiraea alpina Extract in Preparing Anticancer Drugs. Chinese Patent.

[158] Chung I.M., Kalaiselvi K., Sasireka A., Kim S.-H., Prabakaran M. (2019). Anticorrosive property of *Spiraea cantoniensis* extract as an eco-friendly inhibitor on mild steel surface in acid medium. J. Dispers. Sci. Technol..

[159] Pavlović P., Mitrović M., Djurdjević L., Gajić G., Kostić O., Bojović S. (2007). The ecological potential of *Spiraea van-hauttei* (Briot.) Zabel for urban (The city of Belgrade) and fly ash deposit (Obrenovac) landscaping in Serbia. Pol. J. Environ. Stud..

[160] Sun Y., Li L., Wang Y., Dai X. (2020). Morphological and physiological responses of *Spiraea* species to saline water irrigation. Am. Soc. Hort. Sci..

[161] Jin Z., Zhang J. (2017). Analysis of volatile components from *Spiraea trilobata* L. leaves using HS-SPME and GC-MS. J. Shanxi Agr. Sci..

[162] Yan A.J., Xing Y., Yue X. (2011). Analysis and comprehensive evaluation of chemical composition of *Spiraea pubescens*. J. Arid. Res. Environ..

[163] Yu Y., Bi L., Liu H., Shi X., Zhao L. (2012). Study on blanching technology of *Spiraea* tea. Food. Mach..

